# Development of a risk factor framework to inform machine learning prediction of young people’s mental health problems: a Delphi study

**DOI:** 10.1093/jamiaopen/ooaf166

**Published:** 2025-12-23

**Authors:** Katherine Parkin, Ryan Crowley, Rachel Sippy, Shabina Hayat, Yi Zhang, Emily Brewis, Nicole Marshall, Tara Ramsay-Patel, Vahgisha Thirugnanasampanthan, Guy Skinner, Peter Fonagy, Carol Brayne, Anna Moore

**Affiliations:** Department of Public Health and Primary Care, University of Cambridge, Cambridge CB2 0SR, United Kingdom; Department of Psychiatry, University of Cambridge, Cambridge CB2 0SZ, United Kingdom; Cambridge Public Health, University of Cambridge, Cambridge CB2 1PZ, United Kingdom; NYU Grossman School of Medicine, New York University, New York, NY 10016, United States; Department of Psychiatry, University of Cambridge, Cambridge CB2 0SZ, United Kingdom; Department of Behavioural Science and Health, University College London, London WC1E 6BT, United Kingdom; Institute of Epidemiology and Health Care, University College London, London WC1E 7HB, United Kingdom; Department of Psychiatry, University of Cambridge, Cambridge CB2 0SZ, United Kingdom; Department of Psychiatry, University of Cambridge, Cambridge CB2 0SZ, United Kingdom; Department of Public Health and Primary Care, University of Cambridge, Cambridge CB2 0SR, United Kingdom; Division of Psychology and Language Sciences, University College London, London WC1H 0AP, United Kingdom; Department of Psychiatry, University of Cambridge, Cambridge CB2 0SZ, United Kingdom; Department of Public Health and Primary Care, University of Cambridge, Cambridge CB2 0SR, United Kingdom; Division of Psychology and Language Sciences, University College London, London WC1H 0AP, United Kingdom; Department of Psychiatry, University of Cambridge, Cambridge CB2 0SZ, United Kingdom; Cambridge Public Health, University of Cambridge, Cambridge CB2 1PZ, United Kingdom; Department of Psychiatry, University of Cambridge, Cambridge CB2 0SZ, United Kingdom

**Keywords:** mental health, risk prediction, early identification, machine learning, children and young people

## Abstract

**Objectives:**

To create a theoretical framework of mental health risk factors to inform the development of prediction models for young people’s mental health problems.

**Materials and Methods:**

We created an initial prototype theoretical framework using a rapid literature search and stakeholder discussion. A snowball sampling approach identified experts for the Delphi study. Round 1 sought consensus on the overall approach, framework domains, and life course stages. Round 2 aimed to establish the points in the life course where exposure to specific risk factors would be most influential. Round 3 ranked risk factors within domains by their predictive importance for young people’s mental health problems.

**Results:**

The final framework reached consensus after 3 rounds and included 287 risk factors across 8 domains and 5 life course stages. Twenty-five experts completed round 3. Domains ranked as most important were “Social and Environmental” and “Psychological and Mental Health.” Ranked lists of risk factors within domains and heat maps showing the salience of risk factors across life course stages were generated.

**Discussion:**

The study integrated multidisciplinary expert perspectives and prioritized health equity throughout the framework’s development. The ranked risk factor lists and life stage heat maps support the targeted inclusion of risk factors across developmental stages in prediction models.

**Conclusion:**

This theoretical framework provides a roadmap of important risk factors for inclusion in early identification models to enhance the predictive accuracy of childhood mental health problems. It offers a useful theoretical reference point to support model building for those without domain expertise.

## Introduction

Mental health problems among UK children are rising, with 20.3% of 8-16 year olds having a probable mental disorder,^1^ mirroring trends in the West.^2–4^ Approximately half of all mental health problems begin by age 14, and three-quarters by 24,^5^ often causing lifelong impacts on education, relationships, lifestyle behaviors, and employment opportunities.^6–9^ Barriers to accessing child and adolescent mental health services include stigma, cultural norms, and personal reluctance. Practical issues also play a role, like remote living, caregiver dependence, and academic pressures.^10–12^ These disproportionately affect under-served groups, worsening health inequalities.^13^ By 2040, anxiety and depression are projected to be among the 3 disorders with the greatest increase in prevalence in the most deprived areas, making them major contributors to health inequity.^14^

Currently, mental health problems are largely identified through specialist assessment.^15^ Young people or their caregivers may seek help via health, social care, or school referrals;^16^ however, long waiting lists delay specialist care. Early identification is therefore critical, and clinical prediction tools could help by facilitating early detection and directing individuals to timely support.^17–19^

Existing psychiatric prediction models have major limitations. Although some have shown promise in research, few are used clinically.^20^^,^^21^ A common barrier is limited interpretability,^22^ as “black-box” models lack transparency, reducing clinicians’ trust in them.^23^^,^^24^ Furthermore, biased data can worsen health disparities,^25–27^ making bias mitigation essential throughout the data pipeline.

Effective psychiatric prediction models require a priori predictor selection based on evidence or domain expertise to minimize bias and enhance clinical utility.^28^ To support the development of prediction models for identifying childhood mental health problems, we created a theoretical risk factor framework using a Delphi methodology—an iterative expert consensus approach allowing reflective input and nuanced outputs.^29^ Unlike focus groups or single interviews, the Delphi method allows structured refinement. This method has been leveraged in the fields of public health and psychiatry, including to seek expert opinion on complex questions, develop conceptual frameworks, and inform prioritization efforts.^30–34^ To our knowledge, no such childhood mental health framework currently exists to specifically inform prediction model development.

Overall, the aims of this study were to:

- gain feedback on the validity of a prototype theoretical framework of risk factors for young people’s mental health problems;
- identify any key risk factors absent from the framework;
- create a prioritized list of risk factors to guide data extraction and future model development;
- capture a diverse range of expert perspectives to mitigate health inequalities potentially exacerbated by the study and subsequent early identification tools.

## Methods

### Development of the initial framework

We performed a rapid literature review to identify youth mental health risk factors—defined as variables predictive of, or associated with, mental health problems. For inclusion in our prototype framework, factors had to originate from moderate/high-quality studies and demonstrate a moderate/large effect size, as defined by the source paper.

Risk factors suggested by clinicians and other professionals were included during the framework’s initial development, combining literature review and professional consultation for the Delphi study. Later rounds incorporated further literature and participant-suggested risk factors (see Supplementary File 1 for references). Candidate risk factors were organized into domains through an iterative process. Initially structured around the biopsychosocial model, domains were refined—added to or collapsed—based on the identification of new factors and ongoing discussion between the research team and clinicians. Life course stages were also established to determine each factor’s temporal relevance across development.

### Identifying experts in the field

For round 1 recruitment, we conducted stakeholder mapping and snowball sampling to identify multidisciplinary experts, starting with known experts who then nominated others in the field. Round 2 employed purposive sampling to fill representation gaps with respondents suggesting colleagues from underrepresented areas, supplemented by independent searches through academic networks. We also broadened geographic diversity by reaching out to international experts. Respondents reported their roles and years of experience.

### Running the survey

The Delphi study was conducted over 3 rounds, each focusing on a specific component of framework refinement. Data collection took place between June 2021 and May 2022 (round 1: June-August 2021; round 2: October-November 2021; round 3: April-May 2022). For questions requiring group consensus, a predefined threshold of a median score of 70% was applied. If this threshold was not met, modifications were made to the framework, and the revised item was reevaluated in the subsequent round.

Round 1 aimed to establish consensus on the overall approach, domains, and life course stages, with participants rating their agreement on a 1-10 scale. Round 2 focused on ranking the domains in terms of predictive utility for mental ill-health, and identifying the life stages at which exposure to each risk factor would be most predictive of developing mental health problems. Participants also suggested any missing risk factors and reviewed round 1 revisions. Round 3 involved ranking risk factor importance within domains and also assessing when exposure to newly introduced risk factors from round 2 would be most consequential.

**Figure 1.**
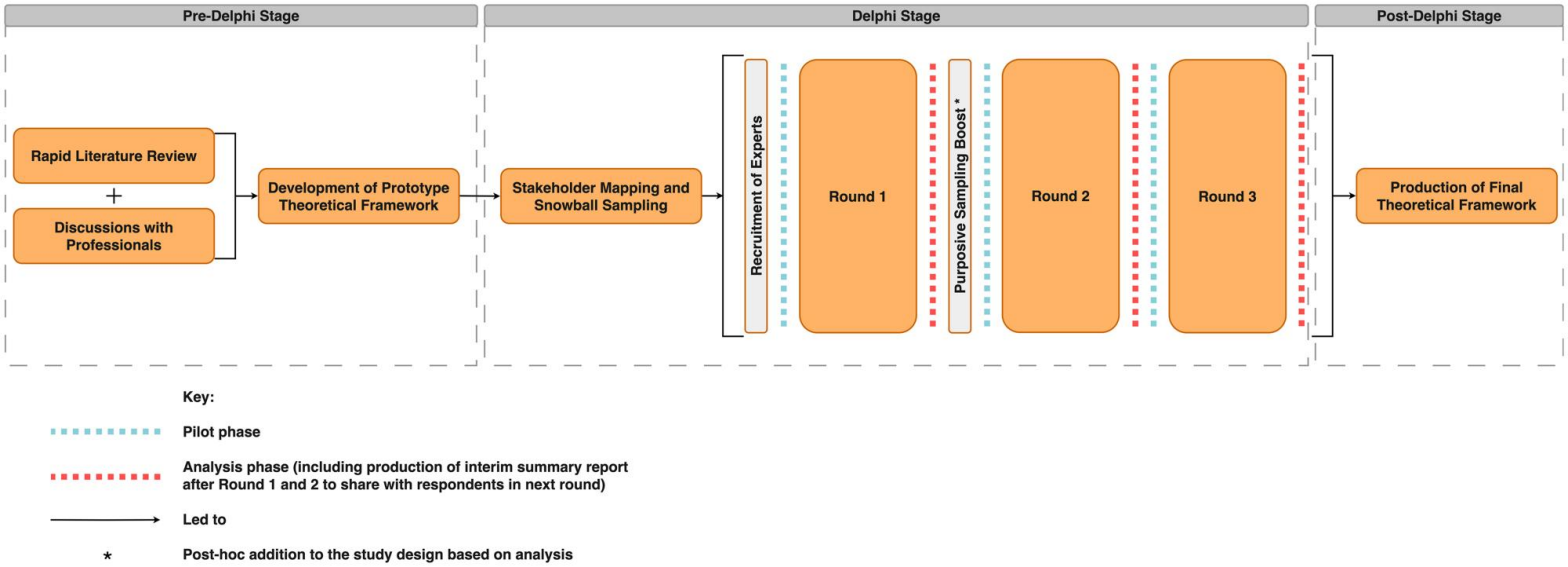
Flow diagram of study stages.

Each round was piloted by 2 external reviewers to identify usability issues. Participants were given several weeks to respond to each round, with deadline reminders. Rounds 1 and 2 were administered using the REDCap platform,^35^^,^^36^ while round 3 was hosted on Qualtrics XM^37^ due to functionality requirements. The overall structure of the Delphi process is illustrated in Figure 1.

### Domain ranking (rounds 2 and 3)

Respondents ranked 8 domains by predictive importance for childhood mental health problems, viewing only the domains they indicated they could evaluate. Median scores determined the overall importance across respondents.

### Life course stage heat maps (rounds 2 and 3)

Respondents indicated relevant life course stages for risk factors within their assessed domains. Heat maps were generated to visualize critical exposure periods, indicating when exposure to each risk factor would be most predictive of mental ill-health.

### Risk factor ranking (round 3)

Respondents rated the importance of each risk factor for mental ill-health prediction on a 0-100 scale using a slider, where “0” indicated no importance and “100” indicated highest importance. First, participants selected the domains they felt able to assess, and then rated their level of expertise in each selected domain using a 0-4 scale (0 = none; 1 = basic; 2 = intermediate; 3 = advanced; 4 = expert). Final rankings included only responses from participants who self-rated as having at least intermediate expertise in the domain. Respondents could indicate if they felt unable to assess a factor.

To calculate the rank of each risk factor within its domain, we: (1) summed the total points each respondent allocated across all rated factors; (2) determined the proportion of points assigned to each individual risk factor; and (3) calculated the mean score across all eligible respondents. These means were used to generate prioritized lists of risk factors within each domain. Importantly, factor rankings were not comparable across domains, as different participants contributed to each set.

### Ethical approval

The study was reviewed and approved by the University of Cambridge Psychology Research Ethics Committee (REC Ref: PRE.2021.028).

### Additional methodological details

For more detailed methodology, see chapter 4 of the lead author’s thesis.^38^

## Results

### Initial model framework

Following the initial literature review, 193 risk factors were extracted from 14 systematic reviews and 2 independent policy or research reports. Prior to incorporating suggestions from survey respondents, the preliminary domains were: Environmental (including exposures); Social (including safeguarding and adverse childhood experiences); Biological (nonpathological); Behavioral; Educational; Clinical (physical health); Clinical (psychological or mental health); and Patterns of Service Use. The original life course stages were: Parental; Pregnancy; Birth and Infancy (0-1 years); and Childhood to Early Adulthood (1-25 years).

### Participant recruitment and demographics

A total of 228 individuals were invited to participate in round 1, of whom 82 agreed. Among these, 50 completed the online consent form. Of those who consented, 39 completed at least 1 survey item beyond consent or demographic questions, and were counted as participants in round 1.

Demographic analysis of round 1 revealed underrepresentation of experts from ethnic minority backgrounds and from the field of genetics. To address these gaps, purposive sampling was applied in round 2, yielding 55 completed responses.

No new participants were recruited for round 3; invitations were issued to those who had participated in round 2. Of these, 29 individuals consented, and 25 completed round 3. Demographic characteristics of respondents from rounds 1 and 2 are presented in Table 1.

**Table 1.** Demographics of individuals from rounds 1 and 2 (ie, pre- and post-purposive sampling) who completed at least 1 part of the survey.

		No. of respondents in round 1 (%)	No. of respondents in round 2 (%)	**Mean years of experience in round 2, SD** ^a^
Gender	Men	13 (33.33)	13 (23.64)	24.8, SD = 12.6
	Women	26 (66.67)	33 (60.00)	19.3, SD = 10.1
	Nonbinary/third gender	0 (0.00)	1 (1.82)	7.0, SD = N/A
	Prefer not to say	0 (0.00)	1 (1.82)	10.0, SD = N/A
	Unknown/missing gender	0 (0.00)	7 (12.73)	N/A, SD = N/A
Ethnicity	White English, Welsh, Scottish, Northern Irish, or British Irish	27 (69.23)	29 (52.73)	21.6, SD = 11.2
	White and Asian	1 (2.56)	0 (0.00)	N/A, SD = N/A
	Other White	10 (25.64)	14 (25.45)	21.3, SD = 11.9
	Indian	1 (2.56)	2 (3.64)	11.0, SD = 1.41
	Pakistani	0 (0.00)	1 (1.82)	21.0, SD = N/A
	Other Asian	0 (0.00)	1 (1.82)	10.0, SD = N/A
	Any other mixed or multiple ethnic group	0 (0.00)	1 (1.82)	7.0, SD = N/A
	Unknown/missing ethnicity	0 (0.00)	7 (12.73)	N/A, SD = N/A
Sector^b^	Academia	18 (46.15)	19 (34.55)	20.2, SD = 12.0
	Clinical	18 (46.15)	23 (41.82)	20.1, SD = 9.53
	Nonclinical professional	2 (5.13)	3 (5.45)	19.0, SD = 18.2
	Education (teachers)	4 (10.26)	4 (7.27)	22.0, SD = 5.89
	Other	4 (10.26)	4 (7.27)	26.5, SD = 17.4
	Unknown/missing sector	1 (2.56)	11 (20.00)	N/A, SD = N/A
Specialty^b^	Behavioral research	3 (7.69)	3 (5.45)	16.7, SD = 5.77
	Biomarkers research	1 (2.56)	1 (1.82)	20.0, SD = N/A
	Child and adolescent psychiatry	12 (30.77)	14 (25.45)	17.8, SD = 11.8
	Child and adolescent psychology	17 (43.59)	18 (32.73)	18.4, SD = 9.78
	Child development	4 (10.26)	4 (7.27)	18.0, SD = 6.27
	Child maltreatment/trauma	8 (20.51)	8 (14.55)	23.5, SD = 9.72
	Child psychopathology	7 (17.95)	7 (12.73)	20.4, SD = 9.13
	Social sciences	2 (5.13)	2 (3.64)	13.5, SD = 4.95
	Genetics	1 (2.56)	1 (1.82)	11.0, SD = N/A
	Education	8 (20.51)	8 (14.55)	21.5, SD = 11.8
	Social care	4 (10.26)	3 (5.45)	22.0, SD = 12.5
	Other local authority services	1 (2.56)	1 (1.82)	20.0, SD = N/A
	Pediatrics	3 (7.69)	4 (7.27)	17.9, SD = 7.94
	Policy	1 (2.56)	1 (1.82)	21.0, SD = N/A
	Prevention	3 (7.69)	2 (3.64)	17.0, SD = 5.66
	Public health	1 (2.56)	1 (1.82)	21.0, SD = N/A
	Other	3 (7.69)	6 (10.91)	22.2, SD = 15.7
	Unknown/missing specialty	0 (0.00)	10 (18.18)	N/A, SD = N/A
Role^b^	Administrative	0 (0.00)	0 (0.00)	N/A, SD = N/A
	Management	5 (12.82)	8 (14.55)	20.9, SD = 5.08
	Clinician	17 (43.59)	21 (38.18)	21.4, SD = 11.0
	Researcher	19 (48.72)	21 (38.18)	20.0, SD = 11.4
	Support staff	0 (0.00)	0 (0.00)	N/A, SD = N/A
	Trained professional	9 (23.08)	10 (18.18)	24.2, SD = 13.6
	Health front line	3 (7.69)	6 (10.91)	19.4, SD = 5.41
	Other	1 (2.56)	1 (1.82)	20.0, SD = N/A
	Unknown/missing role	0 (0.00)	10 (18.18)	N/A, SD = N/A

a Average years of experience and statistics calculated from those who completed at least 1 part of the questionnaire.

b Participants could tick more than 1 specialty; thus, results add up to more than the sample size.

### Delphi round 1 findings

In round 1, the mean professional experience among respondents was 21.24 years. Of the 39 respondents, 26 (66%) identified as female and 13 (34%) as male. In terms of ethnicity, 71% identified as White British, 24% as Other White, 3% as Indian, and 3% as White and Asian. Regarding their primary work sector, 46% were in academia, 46% in clinical roles, 5% were nonclinical professionals, and 10% in the education sector; respondents could report working across multiple sectors. Demographic distributions by sex and primary work sector remained broadly consistent across study rounds. Following purposive sampling in round 2, representation from underrepresented ethnic groups improved slightly, although the inclusion of experts in genetics did not.

Most respondents in round 1 agreed with the overall study approach, describing it as clear, well organized, inclusive, and engaging. However, limitations in the original framework were identified, particularly the poor representation of risk factors related to inequalities and the lack of visibility of interactions between individual, societal, and familial influences. Table S1 summarizes the consensus outcomes from round 1. While consensus was reached on all included domains, the proposed life stage grouping of 1-25 years was seen as too broad. Neither the approach to addressing inequalities nor the approach of focusing solely on individual risk factors were deemed acceptable.

Based on round 1 feedback, we implemented several framework changes. We adopted Bronfenbrenner’s ecological model,^39^^,^^40^ restructuring the framework to represent risk factors across 3 levels: individual-, family/caregiver-, and society-level. The life course stages were revised to provide greater specificity and better reflect key developmental transitions (eg, schooling and mental health service engagement), and biological factors such as the onset of puberty. The updated life course stages were defined as: Pregnancy; Birth-2 years; 3-11 years; 12-17 years; and 18-25 years. A new domain entitled “Factors Identified to be Particularly Relevant to Under-Served Populations” was also added, incorporating relevant risk factors, while maintaining their original domain placement too. We did not act on feedback suggesting the inclusion of resilience or protective factors, as this fell outside the framework’s practical scope, particularly since many existing variables could function as either risk or protective factors depending on context. Similarly, we chose not to incorporate networks or interactions between risk factors at this stage.

### Delphi rounds 2 and 3 findings

The revised framework achieved consensus, with the new structure receiving a median rating of 8.0/10 from 42 respondents, while the updated life stages scored 9.0/10. Qualitative feedback about the altered structure was positive. One respondent noted, “The new structure does a good job to break down the post infant life stages and align them to pathways through the education system.” Another commented, “I think the comments in the narrative about the changed framework and what drove it e.g, ecosystemic approach and use of terms like under-served is really welcome and really strengthens it.”

Round 2 achieved consensus on addressing inequalities, with the inclusion of a dedicated domain for under-served populations, which received a median score of 8.0/10. One respondent remarked, “Really useful category. I am pleased to see that the study is addressing these social discrimination factors for under-served populations living in areas with disparity of opportunities and inequitable distribution of resources.” Domain rankings from round 2 are summarized in Table 2 (1 = most important, 8 = least important). These rankings were confirmed in round 3, maintaining consensus among the 25 respondents and a median score of 8.0/10.

**Table 2.** Ranked position of each domain (in terms of importance for mental health prediction).

Rank	Median ranking	Domain
1st	1	Social and Environmental
2nd	2	Psychological and Mental Health
Tied 3rd	4	Behavioral
Tied 3rd	4	Biomarkers
Tied 3rd	4	Education and Employment
Tied 6th	5	Factors Identified to be Particularly Relevant to Under-Served Populations
Tied 6th	5	Physical Health
8th	5.5	Patterns of Service Use

Based on round 2 feedback, the “Factors Identified to be Particularly Relevant to Under-Served Populations” domain was expanded using respondent suggestions and the NIHR INCLUDE guidance.^41^ Respondents also noted varying specificity among risk factors (eg, “experience of past traumatic event” vs “trapped during earthquake”), and some conceptual overlap (eg, “unhealthy diet” and “high levels of ultraprocessed foods in diet”), reflecting source material diversity. However, we deliberately chose not to extrapolate beyond those findings.

**Figure 2.**
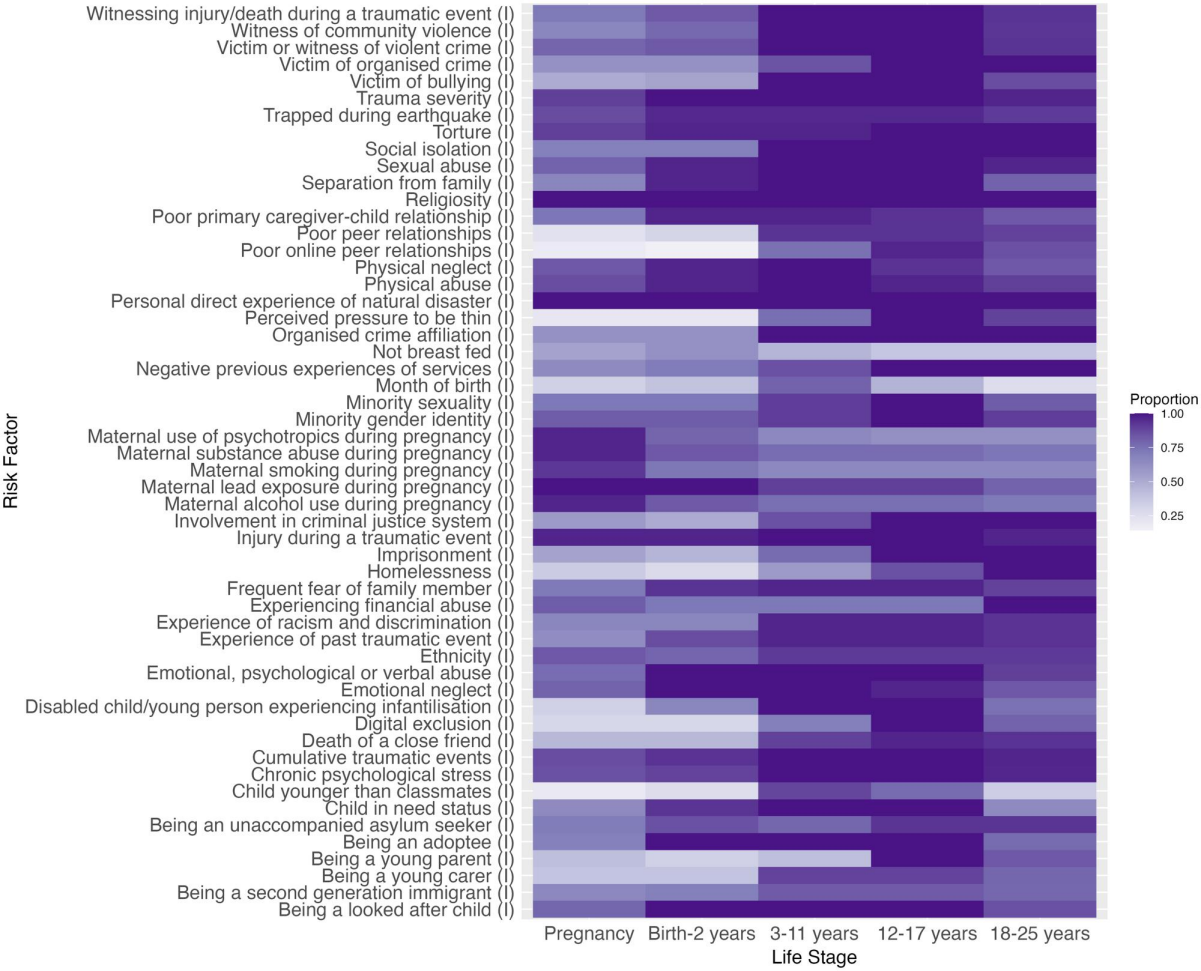
Heat map showing the importance of each risk factor within the Social and Environmental domain (individual factors) across the life course. Proportion (0.00-1.00): proportion of respondents rating each life stage as important for mental health problem development (0.00 = no respondents; 1.00 = all respondents). Abbreviation: I, individual-level.

**Figure 3.**
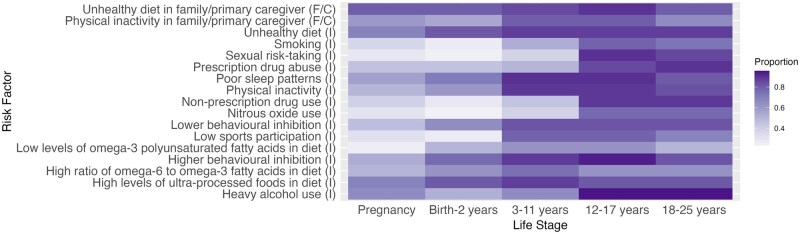
Heat map showing the importance of each risk factor within the Behavioral domain across the life course*. Proportion (0.00-1.00): proportion of respondents rating each life stage as important for mental health problem development (0.00 = no respondents; 1.00 = all respondents). *Some risk factors cannot logically occur at certain life stages. For instance, the individual-level risk factor “smoking” is not applicable during “Pregnancy.” It is likely that participants selected this stage either via the “important for all life stages” checkbox option or due to misinterpreting the risk factor’s level. Abbreviations: I, individual-level; F/C, family/caregiver-level.

**Figure 4.**
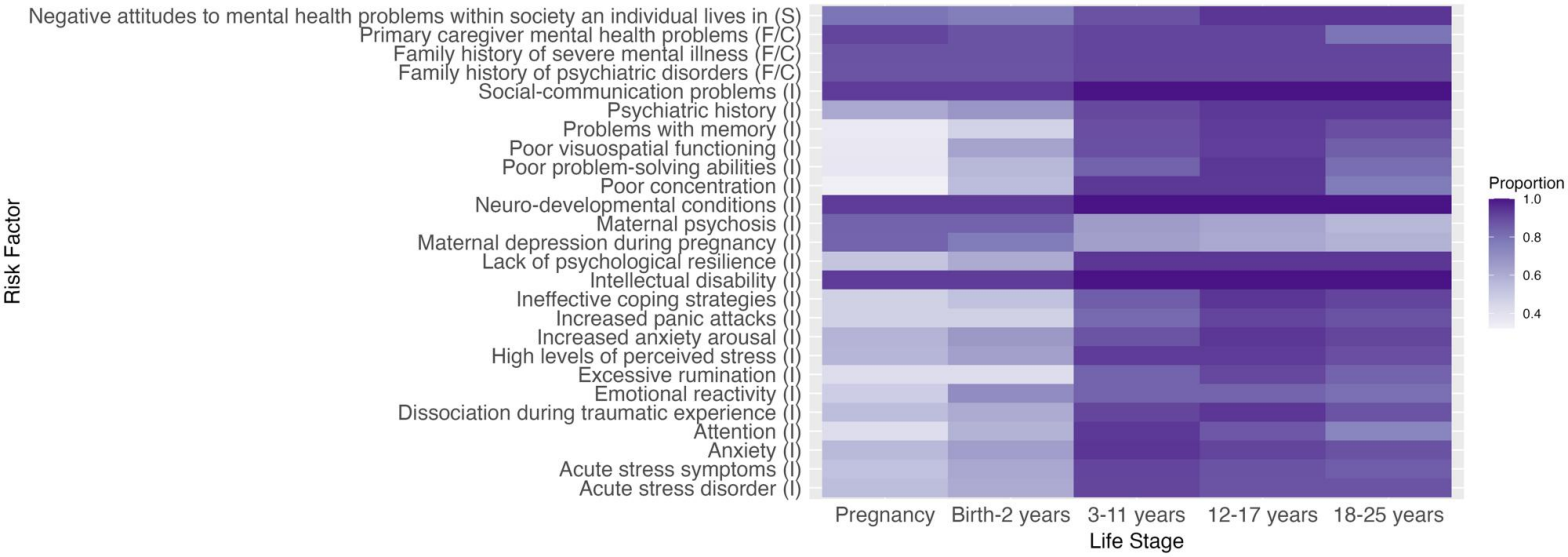
Heat map showing the importance of each risk factor within the Psychological and Mental Health domain across the life course. Proportion (0.00-1.00): proportion of respondents rating each life stage as important for mental health problem development (0.00 = no respondents; 1.00 = all respondents). Abbreviations: I, individual-level; F/C, family/caregiver-level; S, society-level.

In round 3, respondents identified the salient life course stages for newly added risk factors. Combining feedback from rounds 2 and 3, we generated “heat maps” where shading intensity corresponds to the proportion of participants who identified a particular life course stage as important (ie, the critical development periods for each risk factor). Selected heat maps are presented here, with the remainder available in Figures S1-S8. Figure 2 shows the “Social and Environmental” domain (individual-level factors), Figure 3 shows the “Psychological and Mental Health” domain, and Figure 4 shows the “Behavioral” domain.

Figure 2 shows “witnessing injury/death during a traumatic event” and “witness of community violence” were rated as most important during childhood (3-11 years) and adolescence (12-17 years). In contrast, “involvement in the criminal justice system” and “negative previous experience of services” were deemed more important in adolescence (12-17 years) and early adulthood (18-25 years). Maternal factors, including “substance abuse,” “smoking,” and “lead exposure,” were considered most important during pregnancy and infancy (birth-2 years).

Figure 3 illustrates that many behavioral risk factors were considered particularly salient during adolescence and early adulthood. Notably, “sexual risk-taking,” “poor sleep patterns,” “heavy alcohol use,” and “higher behavioral inhibition” at this stage were considered especially relevant to the onset of mental health problems. Some behavioral risk factors, such as “unhealthy diet” and “unhealthy diet in family/primary caregiver,” were rated as important across the life course.

Figure 4 shows that certain psychological and mental health risk factors were consistently rated as important across all life stages, including “intellectual disability,” “social-communication problems,” “family history of severe mental illness,” and “neuro-developmental conditions.” Others, such as “high levels of perceived stress,” “increased anxiety arousal,” and “ineffective coping strategies,” were considered more salient later in development.

### Delphi round 3 findings

**Figure 5.**
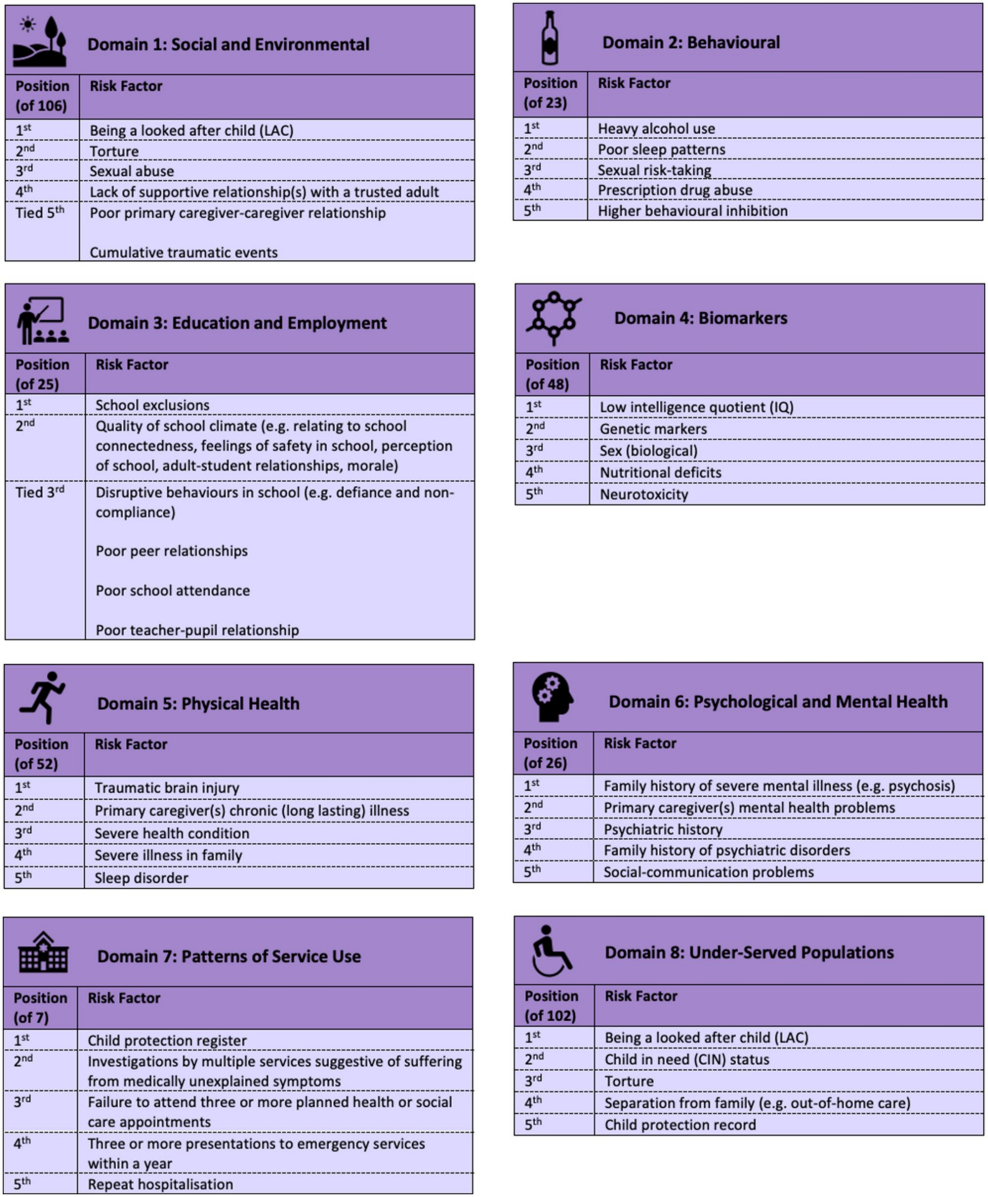
List of most highly ranked risk factors in each domain.

Round 3 feedback led to the production of ordered lists of risk factors within each domain. Table S2 shows respondent expertise by domain. Figure 5 displays each domain’s top-ranked factors, with complete rankings—including number of respondents and confidence intervals—in Tables S3-S10.

These top-ranked factors reflect diverse mental ill-health predictors. “Being a looked after child” was ranked highest in both the “Social and Environmental” and “Under-Served Populations” domains. “Heavy alcohol use” ranked highest among behavioral risk factors, while “school exclusions” topped the “Education and Employment” domain. “Low IQ” was the top-ranked risk factor in the “Biomarkers” domain, and “family history of severe mental illness” was the most highly rated in the “Psychological and Mental Health” domain.

The framework underwent substantial changes, and the main changes are summarized in Table 3. The final framework can be found in Supplementary File 2.

**Table 3.** Summary of main changes to framework over 3 rounds.

Type of change	Description of change
Ecological levels	Added levels (“individual,” “family/caregiver”, and “society level”) to the domains, indicating how risk factors act on an individual
Domain groupings	Refined the domains and their names
Life course groupings	Refined the life course stages by making them narrower
Mitigating health inequalities	Added a domain specifically for those from under-served groupings, taking risk factors from the other 7 domains to highlight factors particularly salient to these populations
Changes to risk factors	Added missing risk factors (resulting in a final list of 287 risk factors), and combined and renamed risk factors where appropriate
Risk factor ranking	Ranked the risk factors within domains in order of importance for predicting mental health problems
Life course stages	Added life course stages to indicate where in the life course risk factors are particularly salient

## Discussion

This Delphi process refined a framework of risk factors for inclusion in early identification models by incorporating diverse perspectives from experts across fields including academia, clinical practice, education, and social care. Starting with a literature-based prototype, iterative rounds of expert feedback strengthened the framework’s robustness. This process aligned with recommended practices for minimizing model bias, particularly the a priori selection of predictors, grounded in clinical experience and empirical evidence.^28^

Health inequality mitigation was prioritized throughout the framework’s development, addressing structural biases that can be embedded at every stage of the data science pipeline.^25^ Equity-focused measures included: targeted literature review; engagement with colleagues from diverse ethnic backgrounds; explicit survey aims; use of NIHR INCLUDE definitions for under-served populations^41^; and a dedicated domain for under-served populations. Retention rates and sample sizes matched comparable studies,^42–45^ with a gender diverse and multidisciplinary expert panel meeting expert input criteria.

The framework evolved considerably in response to the panel’s feedback, including revised domain groupings, more detailed life course stages, and added risk factor levels to contextualize child development within broader ecological systems. This aligns with similar frameworks,^46^^,^^47^ suggesting broad acceptability as a method for structuring risk factors for mental health problems.

The data on risk factor salience across life stages helps with building age-specific prediction models or allows modelers to prioritize risk factors by developmental stage. Since multiple domains were rated as highly important for predicting mental health outcomes, multisource datasets integrating environmental, educational, social, and psychological data could enhance predictive performance. For example, contextual information could be derived from local area indices, educational records, household data linkages, and child-reported or survey-based measures. The strong influence of risk factors in early development suggests predictive models could benefit significantly from incorporating data from birth and early childhood.

Many of the highest-ranked risk factors, such as “being a looked after child” and “family history of severe mental illness,” correspond to well-established Adverse Childhood Experiences (ACEs).^48^ Others, such as “school exclusions” and “traumatic brain injury,” reflect interactions between physical health, social environment, and mental health trajectories. The “Biomarkers” domain ranked relatively highly, yet such data are less often routinely collected. The future integration of biological information into children’s health records could enhance early prediction. A range of initiatives are emerging (eg, NIHR’s DNA, Children + Young People’s Health Resource [aka D-CYPHR],^49^ and UK Research and Innovation’s [UKRI] Adolescent Health Study^50^) but further research and investment are needed to advance this field.

This study has several limitations. First, despite purposive sampling, ethnic diversity in the expert panel remained limited, and certain expertise, such as genetics, remained underrepresented. Second, the framework aimed to inform mental ill-health prediction rather than determine causal mechanisms, making it more suitable for predictive modeling than for guiding prevention or early intervention strategies. Finally, as the framework was developed predominantly by European experts, generalizability to other cultural or geographic contexts may be limited.

### Next steps and future work

The team applied the Delphi-developed risk factor framework to create a prototype early identification model for young people with social care contact.^51^^,^^52^ Initial models show promise but require considerable refinement before clinical implementation as decision support tools. The work has been guided throughout by in-depth consultations with young people and caregivers regarding acceptable use of routine data and machine learning approaches to early identification of mental health problems.^53^ This public consultation continues to inform ongoing development.

Next steps include evaluating the prioritized risk factors in prediction models using linked administrative datasets, and developing natural language processing algorithms to extract relevant information from free-text electronic records.

Future work could expand the framework to incorporate protective factors and interactions, providing a more comprehensive understanding of risk and protective pathways. Additional studies should assess the framework’s generalizability across different health systems, cultures, and socioeconomic contexts, especially beyond Europe.

In conclusion, this Delphi-developed framework provides a roadmap of priority risk factors for childhood mental ill-health prediction models, serving as a practical “wish list” for model building. Its ranked risk factors and life course heat maps guide data collection and variable selection across developmental stages. Unlike black-box models, the framework offers a transparent, interpretable foundation for building equitable prediction tools. Such structured approaches can improve early identification of young people at risk of developing mental health problems.

## Supplementary Material

ooaf166_Supplementary_Data

## Data Availability

The data underlying this article cannot be shared publicly due to consent limitations and participant privacy concerns.
