# Supplementary material for: Development of a risk factor framework to inform machine learning prediction of young people’s mental health problems: a Delphi study
Source: JAMIA Open. 2025 Dec 23;8(6):ooaf166. doi: 10.1093/jamiaopen/ooaf166 (PMC12726920; doi:10.1093/jamiaopen/ooaf166)
Supplement: ooaf166_Supplementary_Data [file ooaf166_supplementary_data.zip › Supplementary File 1 - Supplementary Tables and Figures.docx]

**SUPPLEMENTARY MATERIALS**

In addition to the following supplementary materials, more detailed methods and results can be found in Chapter 4 of the lead author’s thesis, available at: ﻿<https://doi.org/10.17863/CAM.119444>.

*Table S1: Consensus results for each of the areas consulted on in Round 1*

|  |  | **Number of respondents (N)** | **% consensus/mean** |
| --- | --- | --- | --- |
| **Domains** | Environmental (inc. exposures) | 37 | 100.00 |
|  | Social (inc. safeguarding and ACEs**) | 35 | 92.11 |
|  | Behavioural | 36 | 100.00 |
|  | Educational | 38 | 100.00 |
|  | Biological (non-pathological) | 37 | 94.87 |
|  | Clinical (physical health) | 37 | 94.87 |
|  | Clinical (psychological or mental health) | 38 | 97.44 |
|  | Patterns of Service Use | 33 | 91.67 |
| **Life course stage** | Parental | 33 | 89.19 |
|  | Pregnancy | 37 | 100.00 |
|  | Birth-1 year | 36 | 94.74 |
|  | 1-25 years | 36 | 54.29* |
| **Inequalities** |  | 39 | 68.50* |
| **Individual** **risk factors** |  | 39 | 47.90* |

Key: ***** Indicates questions did not reach consensus; ****** **ACEs** = adverse childhood experiences

#### **Heat maps for domains 1-8 showing the importance of each risk factor across the life course**

***Note to the reader regarding all heat maps:*** *some risk factors are logically impossible at certain life course stages; however, all collected data are shown in the heat maps for completeness. (It seems likely these stages were accidentally marked by respondents who ticked the option ‘all life course stages’ to save time).*

*Figure S1a: Heat map showing the importance of each risk factor within the Social and Environmental domain (family/caregiver and society factors) across the life course*


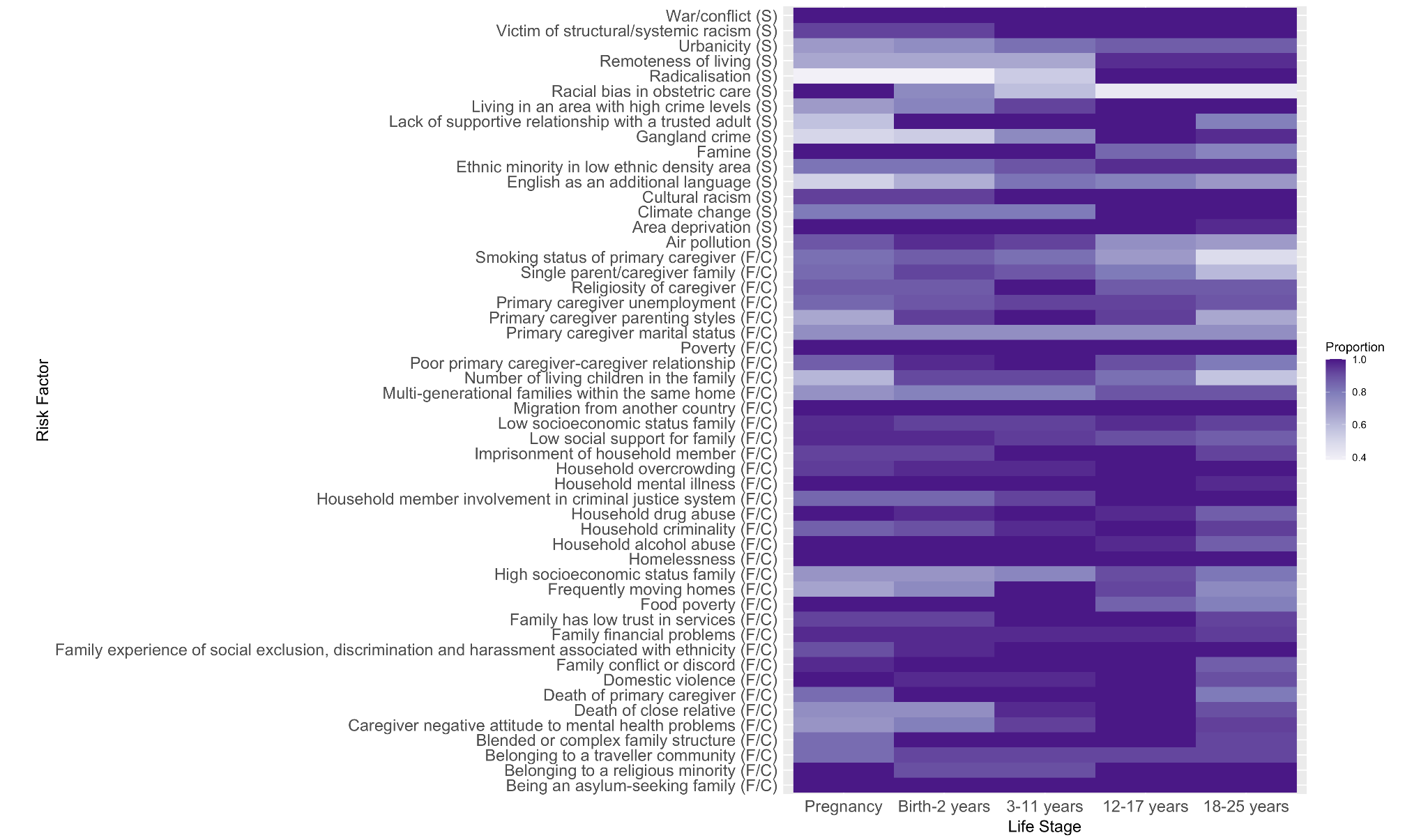


Key: **I** = Individual-level; **S** = Society-level; **F/C** = Family/Caregiver-level. **Proportion (0.00–1.00)** = Proportion of respondents rating each life stage as important for mental health problem development (0.00 = no respondents; 1.00 = all respondents).

Due to the number of risk factors in the Social and Environmental domain, ‘Individual-level’ risk factors are presented separately from the ‘Family/Caregiver-level’ and ‘Society-level’ risk factors.

*Figure S1b: Heat map showing the importance of each risk factor within the Social and Environmental domain (individual factors) across the life course*

*
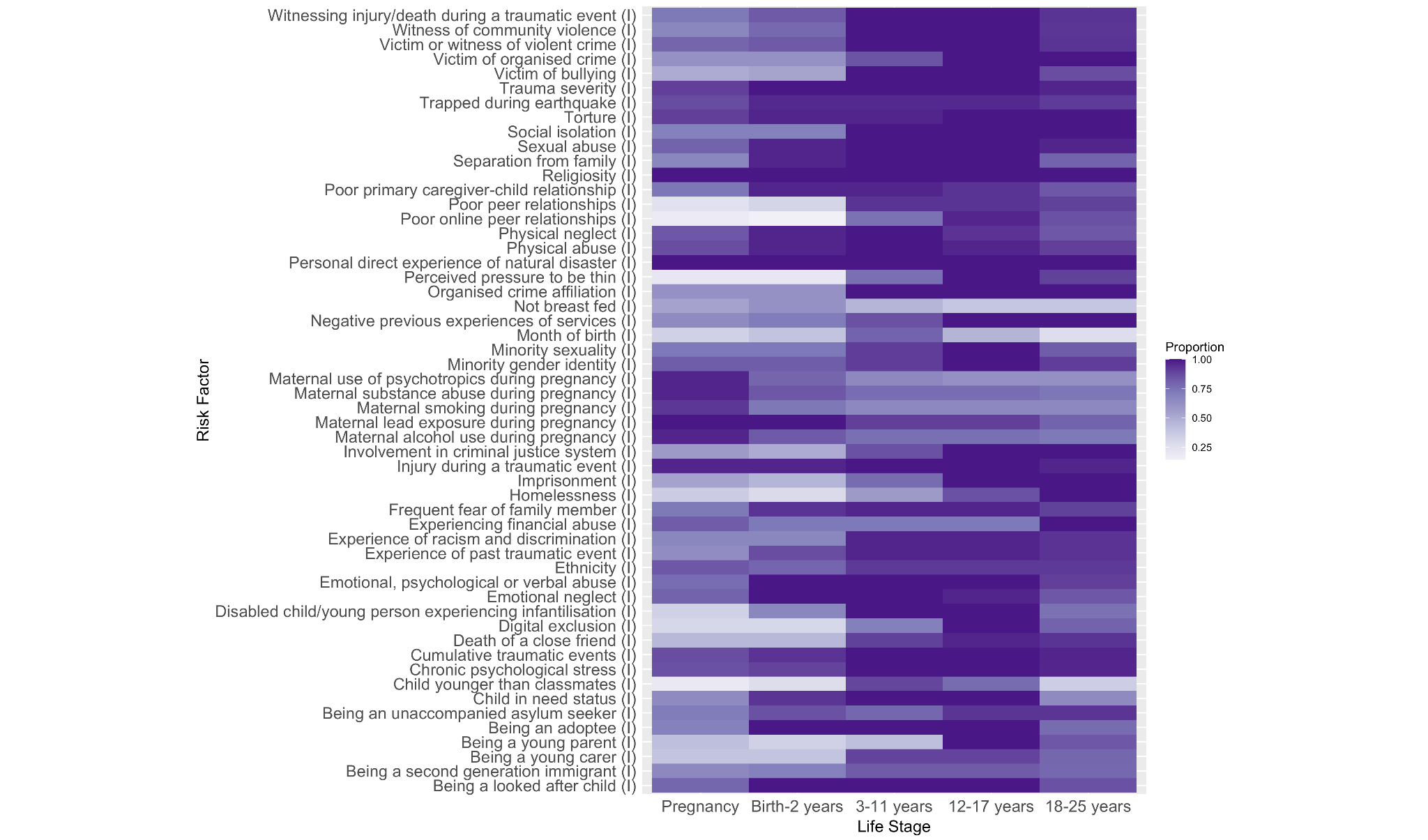
*

Key: **I** = Individual-level; **S** = Society-level; **F/C** = Family/Caregiver-level. **Proportion (0.00–1.00)** = Proportion of respondents rating each life stage as important for mental health problem development (0.00 = no respondents; 1.00 = all respondents).

Due to the number of risk factors in the Social and Environmental domain, ‘Individual-level’ risk factors are presented separately from the ‘Family/Caregiver-level’ and ‘Society-level’ risk factors.

*Figure S2: Heat map showing the importance of each risk factor within the Behavioural domain across the life course*

*
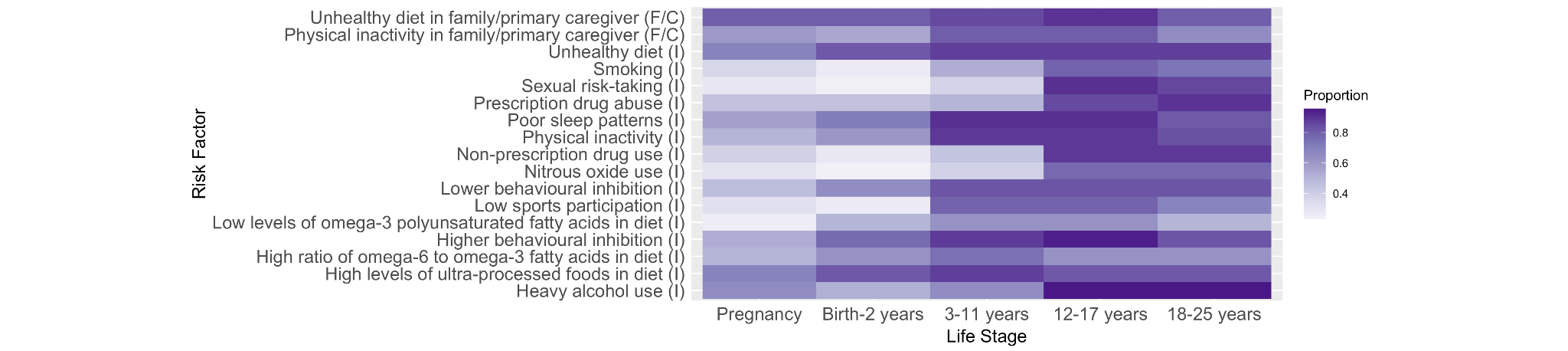
*

Key: **I** = Individual-level; **S** = Society-level; **F/C** = Family/Caregiver-level. **Proportion (0.00–1.00)** = Proportion of respondents rating each life stage as important for mental health problem development (0.00 = no respondents; 1.00 = all respondents).

*Figure S3: Heat map showing the importance of each risk factor within the Education and Employment domain across the life course*

*
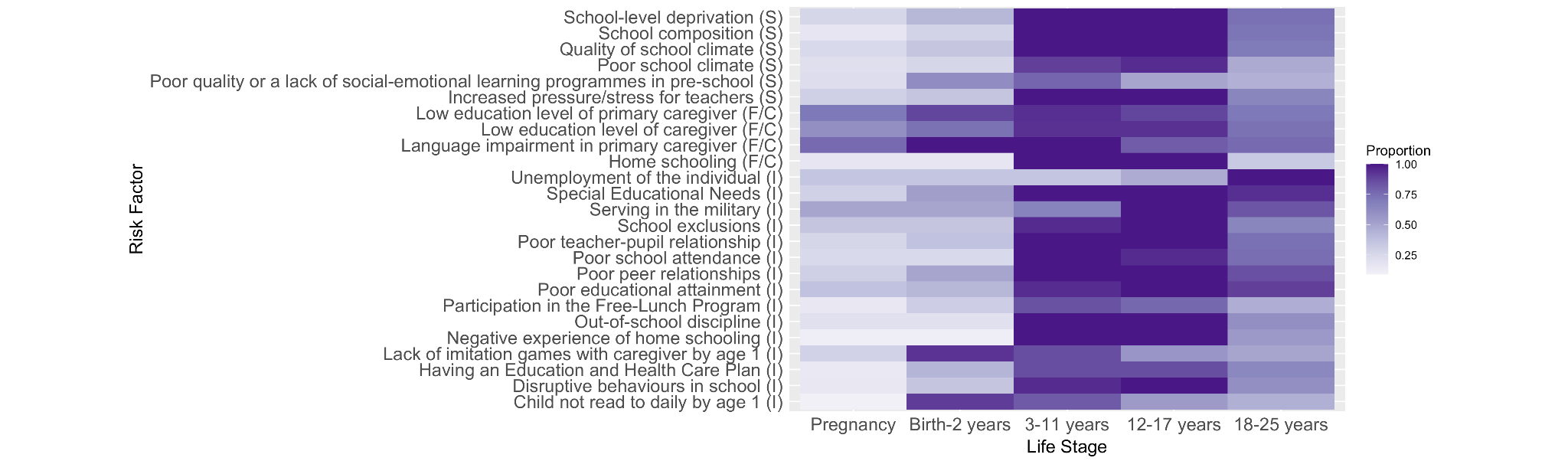
*

Key: **I** = Individual-level; **S** = Society-level; **F/C** = Family/Caregiver-level. **Proportion (0.00–1.00)** = Proportion of respondents rating each life stage as important for mental health problem development (0.00 = no respondents; 1.00 = all respondents).

*Figure S4: Heat map showing the importance of each risk factor within the Biomarkers domain across the life course*

*
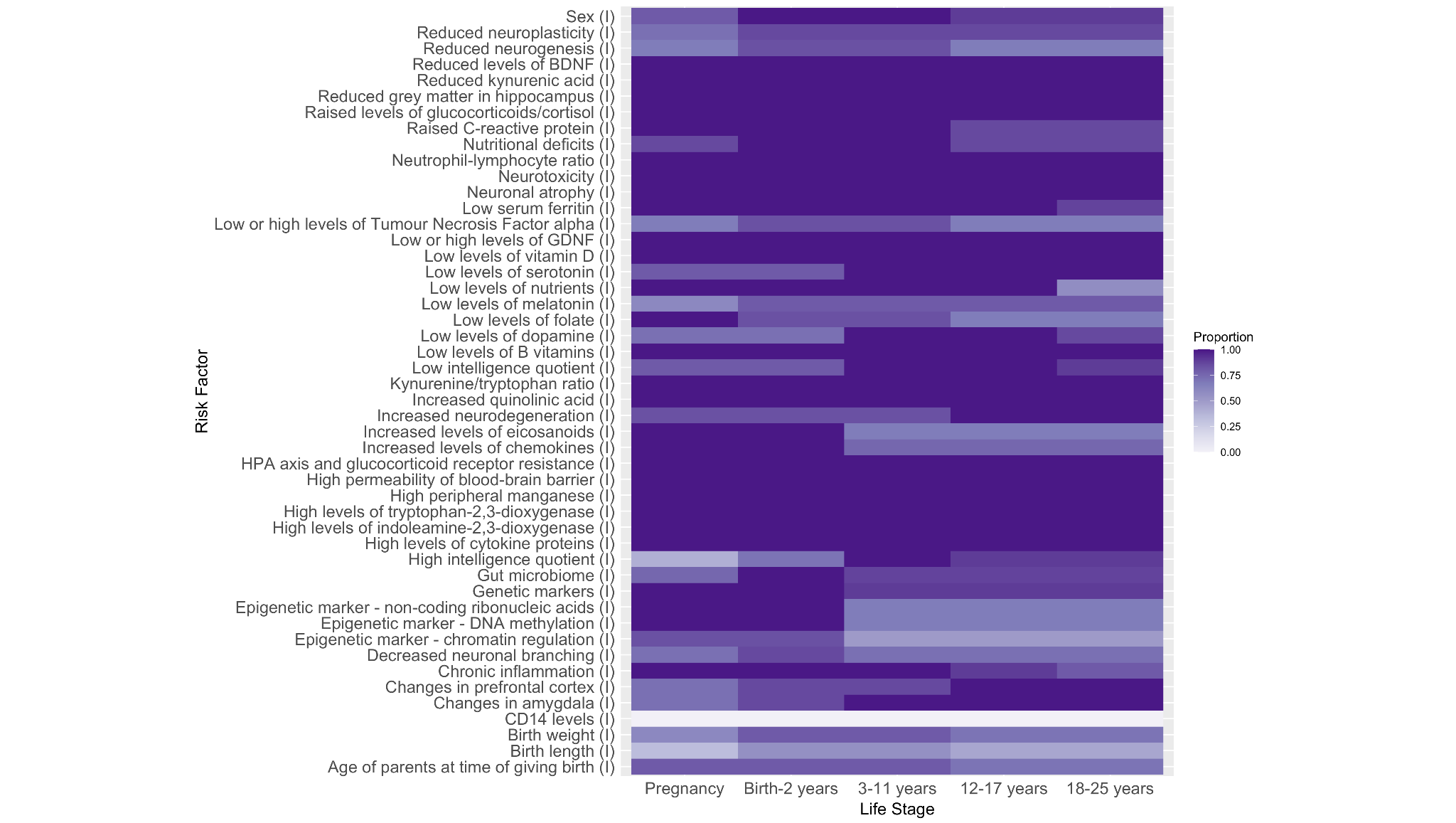
*

Key: **I** = Individual-level; **S** = Society-level; **F/C** = Family/Caregiver-level. **Proportion (0.00–1.00)** = Proportion of respondents rating each life stage as important for mental health problem development (0.00 = no respondents; 1.00 = all respondents).

*Figure S5: Heat map showing the importance of each risk factor within the Physical Health domain across the life course*

*
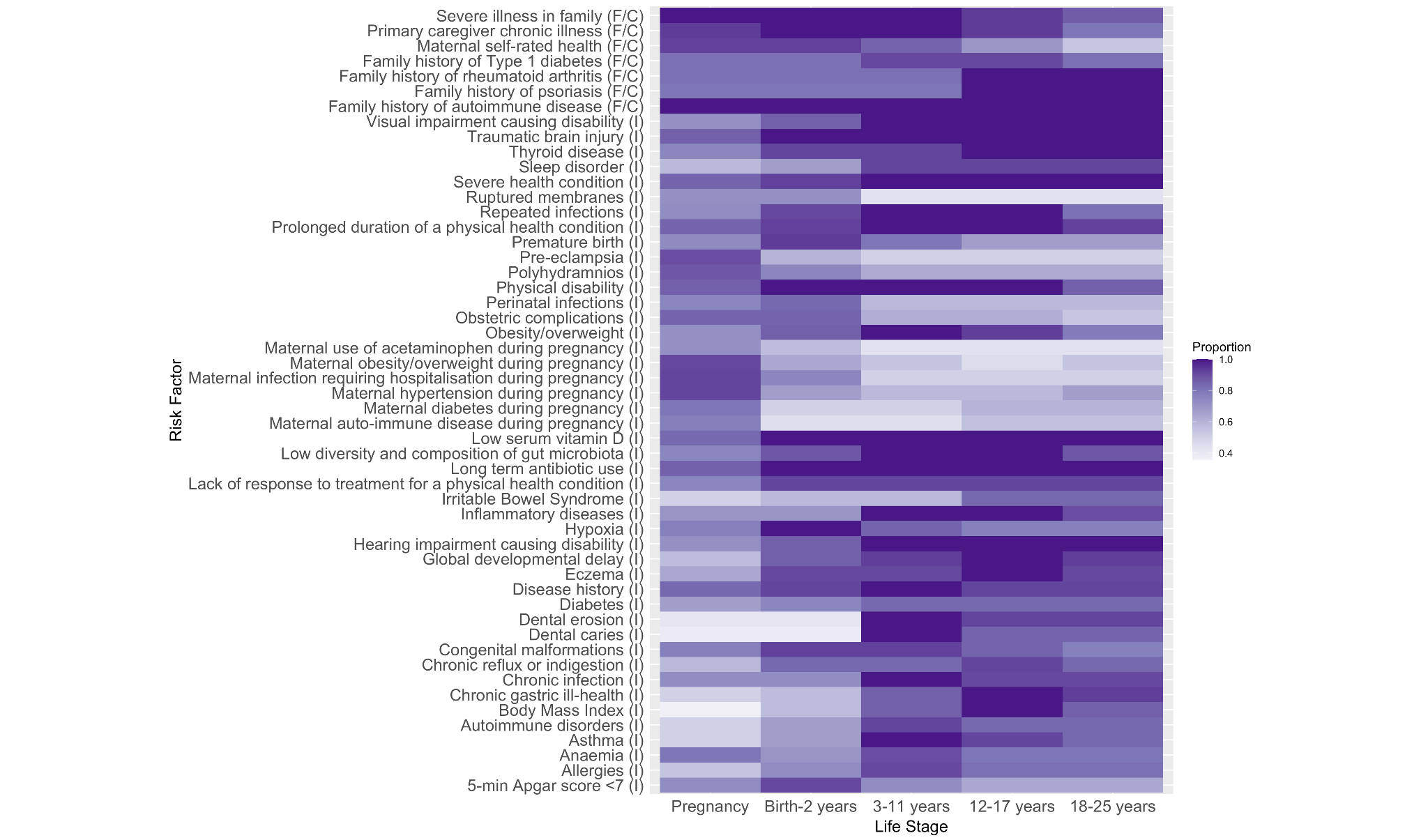
*

Key: **I** = Individual-level; **S** = Society-level; **F/C** = Family/Caregiver-level. **Proportion (0.00–1.00)** = Proportion of respondents rating each life stage as important for mental health problem development (0.00 = no respondents; 1.00 = all respondents).

*Figure S6: Heat map showing the importance of each risk factor within the Psychological and Mental Health domain across the life course*

*
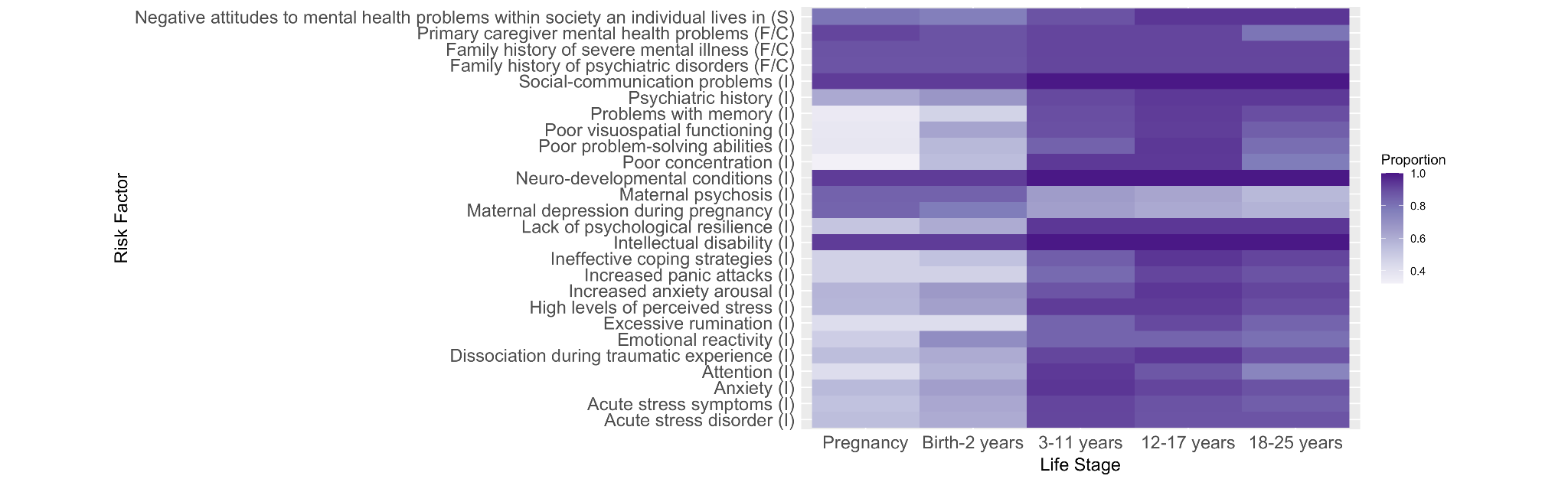
*

Key: **I** = Individual-level; **S** = Society-level; **F/C** = Family/Caregiver-level. **Proportion (0.00–1.00)** = Proportion of respondents rating each life stage as important for mental health problem development (0.00 = no respondents; 1.00 = all respondents).

*Figure S7: Heat map showing the importance of each risk factor within the Patterns of Service Use domain across the life course*

*
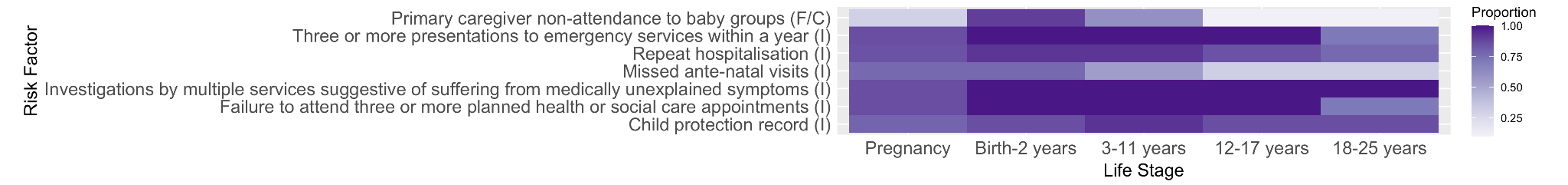
*

Key: **I** = Individual-level; **S** = Society-level; **F/C** = Family/Caregiver-level. **Proportion (0.00–1.00)** = Proportion of respondents rating each life stage as important for mental health problem development (0.00 = no respondents; 1.00 = all respondents).

*Figure S8a: Heat map showing the importance of each risk factor within the Under-Served Population domain (family/caregiver and society factors) across the life course*

*
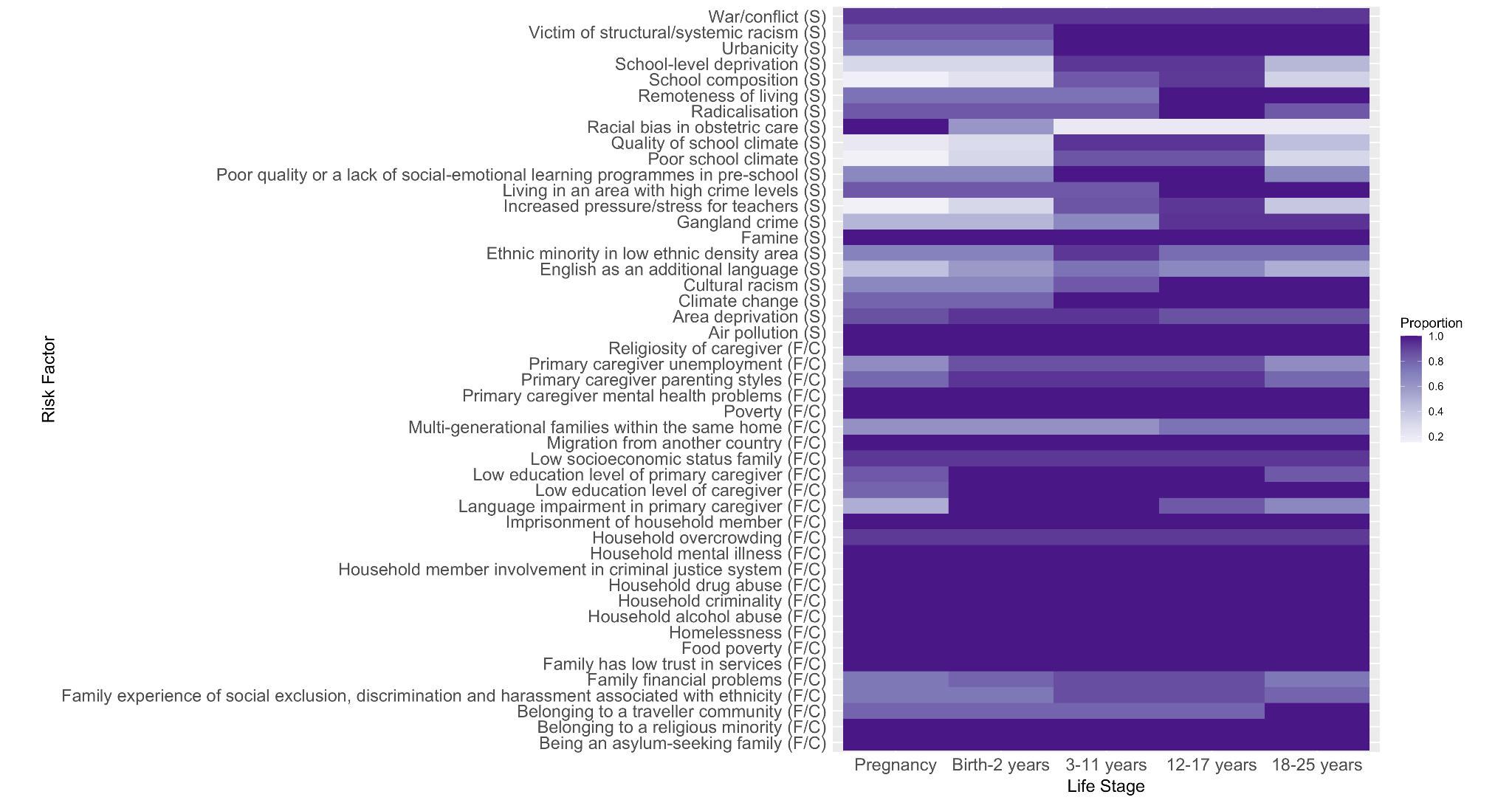
*

Key: **I** = Individual-level; **S** = Society-level; **F/C** = Family/Caregiver-level. **Proportion (0.00–1.00)** = Proportion of respondents rating each life stage as important for mental health problem development (0.00 = no respondents; 1.00 = all respondents).

Due to the number of risk factors in the Under-Served Populations domain, ‘Individual-level’ risk factors are presented separately from the ‘Family/Caregiver-level’ and ‘Society-level’ risk factors.

*Figure S8b: Heat map showing the importance of each risk factor within the Under-Served Populations domain (individual factors) across the life course*

*
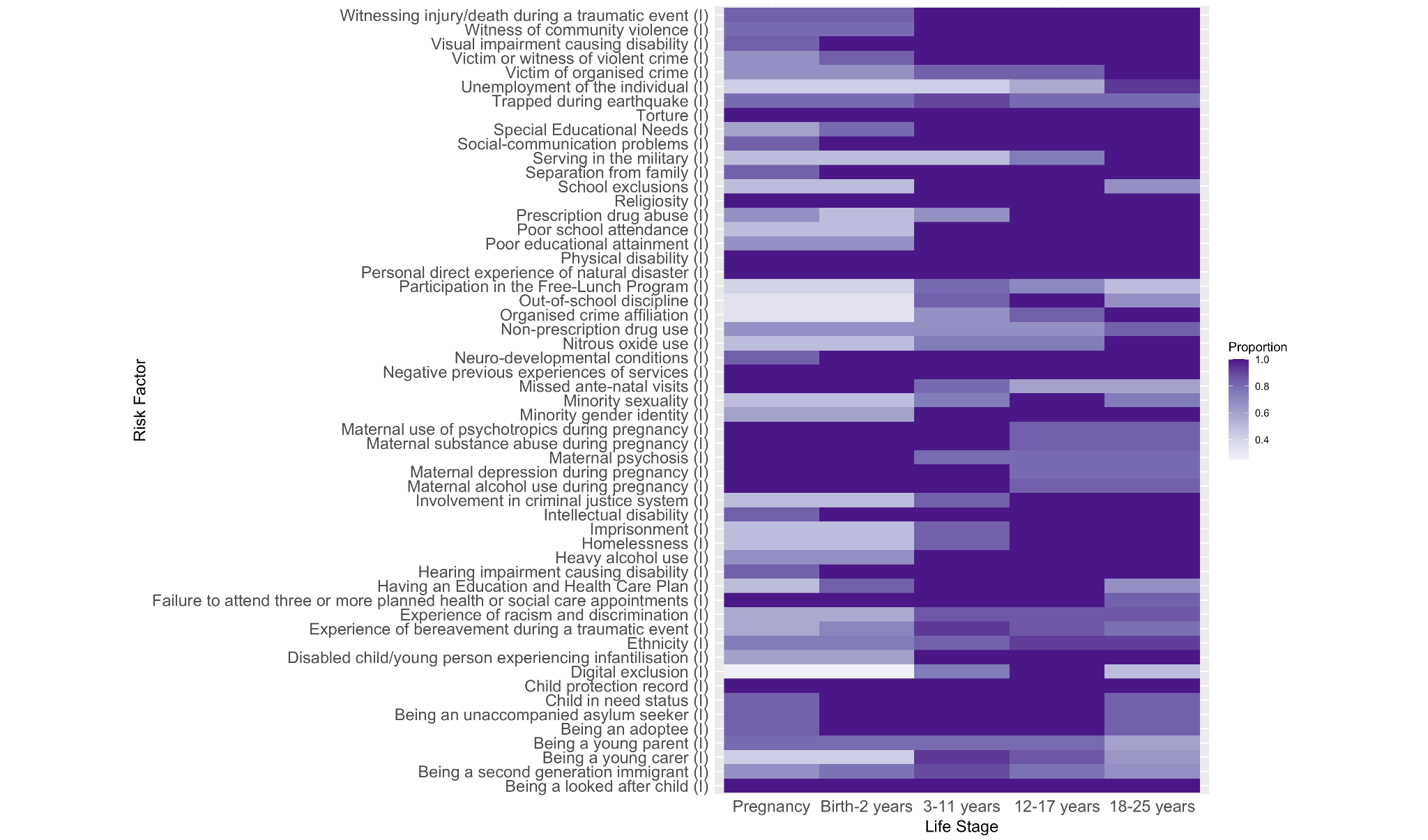
*

Key: **I** = Individual-level; **S** = Society-level; **F/C** = Family/Caregiver-level. **Proportion (0.00–1.00)** = Proportion of respondents rating each life stage as important for mental health problem development (0.00 = no respondents; 1.00 = all respondents).

Due to the number of risk factors in the Under-Served Populations domain, ‘Individual-level’ risk factors are presented separately from the ‘Family/Caregiver-level’ and ‘Society-level’ risk factors.

*Table S2: Number of respondents per domain and their self-rated level of expertise*

| **Domain** | **Number of respondents able to comment (n)** | **Number of respondents unable to comment (n)** | **Percentage of respondents able to comment (%)** | **Expertise Mean [and Standard Deviation]** | **Range of Expertise** |
| --- | --- | --- | --- | --- | --- |
| **Social and Environmental** | 19 | 6 | 76.00 | 2.53 [0.70] | 1 – 4 |
| **Behavioural** | 15 | 10 | 60.00 | 2.93 [0.70] | 2 – 4 |
| **Education and Employment** | 13 | 12 | 52.00 | 2.38 [1.19] | 1 – 4 |
| **Biomarkers** | 7 | 18 | 28.00 | 1.71 [0.95] | 1 – 3 |
| **Physical Health** | 9 | 16 | 36.00 | 2.67 [1.00] | 1 – 4 |
| **Psychological and Mental Health** | 17 | 8 | 68.00 | 3.00 [0.71] | 2 – 4 |
| **Patterns of Service Use** | 9 | 16 | 36.00 | 2.56 [0.53] | 2 – 3 |
| **Factors Identified to be Particularly Relevant to Under-Served Populations** | 7 | 18 | 28.00 | 2.14 [1.21] | 0 – 4 |

Key: Range of expertise: **0** = None; **1** = Basic; **2** = Intermediate; **3** = Advanced; **4** = Expert

**Ranked risk factors within domains 1-8**

*Table S3: Ranked importance of risk factors within domain 1: Social and Environmental*

| **Tied position** | **Rank within domain (out of 106)** | **Risk factor** | **Number of raters (N)** | **Mean score** | **Lower bound (CI)** | **Upper bound (CI)** |
| --- | --- | --- | --- | --- | --- | --- |
|  | 1 | Being a looked after child (LAC) | 14 | 0.014 | 0.010 | 0.025 |
|  | 2 | Torture | 13 | 0.014 | 0.003 | 0.024 |
|  | 3 | Sexual abuse | 16 | 0.014 | 0.012 | 0.023 |
|  | 4 | Lack of supportive relationship(s) with a trusted adult | 15 | 0.014 | 0.011 | 0.024 |
| = | 5 | Poor primary caregiver-caregiver relationship | 16 | 0.013 | 0.008 | 0.020 |
| = | 5 | Cumulative traumatic events | 16 | 0.013 | 0.012 | 0.025 |
| = | 7 | Domestic violence | 16 | 0.013 | 0.011 | 0.023 |
| = | 7 | Trauma severity | 14 | 0.013 | 0.009 | 0.023 |
|  | 9 | Emotional, psychological or verbal abuse | 16 | 0.013 | 0.012 | 0.024 |
|  | 10 | Emotional neglect | 16 | 0.013 | 0.011 | 0.025 |
|  | 11 | Victim of organised crime (e.g. commercial sexual exploitation or via county lines) | 13 | 0.013 | 0.011 | 0.022 |
| = | 12 | Household drug abuse | 16 | 0.013 | 0.010 | 0.021 |
| = | 12 | Poor primary caregiver-child relationship | 16 | 0.013 | 0.010 | 0.024 |
|  | 14 | Physical abuse | 16 | 0.013 | 0.008 | 0.020 |
|  | 15 | Household mental illness | 16 | 0.013 | 0.010 | 0.020 |
|  | 16 | Family conflict or discord | 16 | 0.013 | 0.009 | 0.023 |
|  | 17 | Death of primary caregiver(s) | 16 | 0.013 | 0.009 | 0.020 |
|  | 18 | Caregiver negative attitude to mental health problems | 16 | 0.012 | 0.008 | 0.022 |
|  | 19 | Separation from family (e.g. out-of-home care) | 15 | 0.012 | 0.011 | 0.022 |
| = | 20 | Homelessness | 16 | 0.012 | 0.008 | 0.023 |
| = | 20 | Chronic psychological stress | 16 | 0.012 | 0.004 | 0.021 |
|  | 22 | Household alcohol abuse | 16 | 0.012 | 0.009 | 0.021 |
|  | 23 | Family experience of social exclusion, discrimination and harassment associated with ethnicity | 14 | 0.012 | 0.008 | 0.022 |
|  | 24 | Victim or witness of violent crime | 13 | 0.012 | 0.009 | 0.018 |
|  | 25 | Social isolation | 16 | 0.012 | 0.009 | 0.021 |
|  | 26 | Physical neglect | 16 | 0.012 | 0.003 | 0.021 |
|  | 27 | Witnessing injury/death during a traumatic event | 14 | 0.012 | 0.009 | 0.016 |
|  | 28 | Being an unaccompanied asylum seeker | 15 | 0.012 | 0.005 | 0.021 |
| = | 29 | Being an asylum-seeking family | 14 | 0.012 | 0.008 | 0.017 |
| = | 29 | Victim of bullying | 16 | 0.012 | 0.009 | 0.015 |
| = | 31 | Experience of racism and discrimination | 15 | 0.012 | 0.007 | 0.017 |
| = | 31 | Child in need (CIN) status | 15 | 0.012 | 0.003 | 0.021 |
|  | 33 | Maternal substance abuse during pregnancy | 14 | 0.012 | 0.005 | 0.019 |
|  | 34 | Imprisonment | 14 | 0.012 | 0.009 | 0.021 |
|  | 35 | Experience of past traumatic event | 16 | 0.012 | 0.010 | 0.014 |
|  | 36 | Frequent fear of family member | 16 | 0.012 | 0.004 | 0.022 |
| = | 37 | Involvement in criminal justice system | 15 | 0.012 | 0.009 | 0.015 |
| = | 37 | Homelessness (young person has left home) | 15 | 0.012 | 0.009 | 0.018 |
|  | 39 | War/conflict | 13 | 0.012 | 0.007 | 0.026 |
|  | 40 | Victim of structural/systemic racism | 13 | 0.012 | 0.002 | 0.017 |
|  | 41 | Witness of community violence | 13 | 0.012 | 0.008 | 0.016 |
|  | 42 | Household criminality | 16 | 0.012 | 0.006 | 0.017 |
|  | 43 | Area deprivation (area code) | 15 | 0.011 | 0.007 | 0.025 |
|  | 44 | Injury during a traumatic event | 14 | 0.011 | 0.005 | 0.014 |
|  | 45 | Household member involvement in criminal justice system | 16 | 0.011 | 0.006 | 0.018 |
|  | 46 | Disabled child/young person experiencing infantilisation | 10 | 0.011 | 0.008 | 0.014 |
|  | 47 | Maternal lead exposure during pregnancy | 8 | 0.011 | 0.004 | 0.013 |
|  | 48 | Maternal use of psychotropics during pregnancy | 11 | 0.011 | 0.002 | 0.019 |
|  | 49 | Imprisonment of household member | 14 | 0.011 | 0.002 | 0.015 |
|  | 50 | Maternal alcohol use during pregnancy | 15 | 0.011 | 0.005 | 0.019 |
|  | 51 | Poor peer relationships | 16 | 0.011 | 0.008 | 0.019 |
|  | 52 | Cultural racism | 14 | 0.011 | 0.005 | 0.026 |
|  | 53 | Organised crime affiliation | 12 | 0.011 | 0.007 | 0.013 |
|  | 54 | Death of a close friend | 16 | 0.011 | 0.007 | 0.016 |
|  | 55 | Trapped during earthquake | 11 | 0.011 | 0.002 | 0.012 |
|  | 56 | Family has low trust in services | 15 | 0.011 | 0.003 | 0.022 |
|  | 57 | Famine | 12 | 0.011 | 0.003 | 0.017 |
|  | 58 | Family financial problems | 15 | 0.011 | 0.007 | 0.014 |
|  | 59 | Low social support for family | 16 | 0.011 | 0.008 | 0.015 |
|  | 60 | Being an adoptee | 15 | 0.011 | 0.003 | 0.021 |
|  | 61 | Gangland crime | 13 | 0.011 | 0.003 | 0.016 |
|  | 62 | Being a young carer | 16 | 0.011 | 0.006 | 0.019 |
|  | 63 | Frequently moving homes | 15 | 0.010 | 0.003 | 0.015 |
| = | 64 | Ethnic minority in low ethnic density area | 13 | 0.010 | 0.004 | 0.019 |
| = | 64 | Experiencing financial abuse | 13 | 0.010 | 0.004 | 0.014 |
|  | 66 | Personal direct experience of natural disaster | 16 | 0.010 | 0.003 | 0.013 |
|  | 67 | Household overcrowding | 14 | 0.010 | 0.003 | 0.014 |
|  | 68 | Perceived pressure to be thin | 16 | 0.010 | 0.002 | 0.015 |
|  | 69 | Poverty | 16 | 0.010 | 0.002 | 0.016 |
|  | 70 | Minority gender identity | 11 | 0.010 | 0.003 | 0.014 |
|  | 71 | Food poverty | 13 | 0.010 | 0.004 | 0.013 |
|  | 72 | Poor online peer relationships | 16 | 0.010 | 0.005 | 0.015 |
|  | 73 | Minority sexuality | 12 | 0.010 | 0.003 | 0.014 |
|  | 74 | Death of close relative (excluding primary caregiver(s)) | 16 | 0.010 | 0.002 | 0.015 |
|  | 75 | Being a young parent | 16 | 0.010 | 0.004 | 0.014 |
|  | 76 | Radicalisation | 12 | 0.010 | 0.001 | 0.019 |
|  | 77 | English as an additional language | 13 | 0.010 | 0.002 | 0.016 |
|  | 78 | Racial bias in obstetric care | 11 | 0.009 | 0.001 | 0.018 |
|  | 79 | Maternal smoking during pregnancy | 12 | 0.009 | 0.001 | 0.013 |
|  | 80 | Low socioeconomic status family | 16 | 0.009 | 0.002 | 0.014 |
|  | 81 | Primary caregiver(s) parenting styles (strict/rigid/conventional) | 16 | 0.009 | 0.002 | 0.018 |
|  | 82 | Living in an area with high crime levels | 13 | 0.009 | 0.001 | 0.013 |
|  | 83 | Migration from another country | 14 | 0.009 | 0.002 | 0.011 |
|  | 84 | Remoteness of living | 12 | 0.009 | 0.001 | 0.022 |
|  | 85 | Belonging to a traveller community | 14 | 0.009 | 0.002 | 0.012 |
|  | 86 | Negative previous experiences of services | 16 | 0.009 | 0.005 | 0.016 |
|  | 87 | Belonging to a religious minority | 14 | 0.008 | 0.001 | 0.013 |
|  | 88 | Air pollution | 12 | 0.007 | 0.000 | 0.013 |
|  | 89 | Blended or complex family structure | 16 | 0.007 | 0.003 | 0.019 |
|  | 90 | Primary caregiver(s) unemployment | 16 | 0.007 | 0.001 | 0.009 |
|  | 91 | Digital exclusion | 11 | 0.007 | 0.004 | 0.012 |
|  | 92 | Being a second generation immigrant | 13 | 0.006 | 0.000 | 0.009 |
|  | 93 | Number of living children in the family | 15 | 0.006 | 0.000 | 0.013 |
|  | 94 | Urbanicity | 12 | 0.006 | 0.000 | 0.011 |
|  | 95 | Climate change | 11 | 0.006 | 0.003 | 0.009 |
|  | 96 | Ethnicity | 11 | 0.006 | 0.003 | 0.011 |
|  | 97 | Smoking status of primary caregiver(s) | 13 | 0.006 | 0.001 | 0.011 |
|  | 98 | Multi-generational families within the same home | 13 | 0.005 | 0.000 | 0.008 |
|  | 99 | Religiosity of caregiver(s) | 13 | 0.005 | 0.000 | 0.007 |
|  | 100 | Child younger than classmates | 13 | 0.005 | 0.000 | 0.009 |
|  | 101 | Single parent/caregiver family | 16 | 0.005 | 0.000 | 0.011 |
|  | 102 | Religiosity | 12 | 0.004 | 0.000 | 0.009 |
|  | 103 | Not breast fed | 10 | 0.003 | 0.000 | 0.010 |
|  | 104 | Month of birth | 12 | 0.003 | 0.000 | 0.006 |
|  | 105 | High socioeconomic status family | 15 | 0.003 | 0.000 | 0.007 |
|  | 106 | Primary caregiver(s) marital status | 16 | 0.003 | 0.000 | 0.009 |
|  |  |  | **Range: 8 - 16** | | | |

Key: **CI** = confidence intervals

*Table S4: Ranked importance of risk factors within domain 2: Behavioural*

| **Tied position** | **Rank within domain (out of 23)** | **Risk factor** | **Number of raters (N)** | **Mean score** | **Lower bound (CI)** | **Upper bound (CI)** |
| --- | --- | --- | --- | --- | --- | --- |
|  | 1 | Heavy alcohol use | 14 | 0.085 | 0.047 | 0.187 |
|  | 2 | Poor sleep patterns | 14 | 0.079 | 0.049 | 0.135 |
|  | 3 | Sexual risk-taking | 14 | 0.074 | 0.043 | 0.114 |
|  | 4 | Prescription drug abuse | 11 | 0.069 | 0.025 | 0.173 |
|  | 5 | Higher behavioural inhibition | 10 | 0.059 | 0.036 | 0.112 |
|  | 6 | Non-prescription drug use | 12 | 0.056 | 0.026 | 0.135 |
|  | 7 | Frequent social media use | 14 | 0.053 | 0.029 | 0.083 |
|  | 8 | Physical inactivity | 13 | 0.051 | 0.017 | 0.075 |
|  | 9 | Unhealthy diet in family/primary caregiver(s) | 14 | 0.049 | 0.021 | 0.091 |
|  | 10 | Smoking | 13 | 0.049 | 0.008 | 0.083 |
|  | 11 | Physical inactivity in family/primary caregiver(s) | 14 | 0.048 | 0.015 | 0.097 |
|  | 12 | Nitrous oxide use | 8 | 0.047 | 0.030 | 0.063 |
|  | 13 | High levels of ultra-processed foods in diet | 8 | 0.045 | 0.028 | 0.073 |
|  | 14 | Unhealthy diet | 13 | 0.044 | 0.009 | 0.073 |
|  | 15 | Lower behavioural inhibition | 10 | 0.044 | 0.024 | 0.147 |
|  | 16 | Foods high in trans fats in diet | 10 | 0.040 | 0.007 | 0.077 |
|  | 17 | Excessive sports participation | 12 | 0.039 | 0.016 | 0.072 |
|  | 18 | Low sports participation | 12 | 0.039 | 0.006 | 0.064 |
|  | 19 | Diet low in plant-matter diversity | 8 | 0.039 | 0.003 | 0.057 |
|  | 20 | Excessive physical activity | 12 | 0.038 | 0.016 | 0.133 |
|  | 21 | High ratio of omega-6 to omega-3 fatty acids in diet | 6 | 0.037 | 0.020 | 0.047 |
|  | 22 | Low levels of omega-3 polyunsaturated fatty acids in diet | 6 | 0.036 | 0.019 | 0.048 |
|  | 23 | Diet low in plant-matter | 9 | 0.034 | 0.008 | 0.055 |
|  |  |  | **Range: 6 - 14** | | | |

Key: **CI** = confidence intervals

*Table S5: Ranked importance of risk factors within domain 3: Education and Employment*

| **Tied position** | **Rank within domain (out of 25)** | **Risk factor** | **Number of raters (N)** | **Mean score** | **Lower bound (CI)** | **Upper bound (CI)** |
| --- | --- | --- | --- | --- | --- | --- |
|  | 1 | School exclusions | 7 | 0.056 | 0.049 | 0.105 |
|  | 2 | Quality of school climate (e.g. relating to school connectedness, feelings of safety in school, perception of school, adult-student relationships, morale) | 6 | 0.053 | 0.037 | 0.074 |
| = | 3 | Disruptive behaviours in school (e.g. defiance and non-compliance) | 7 | 0.052 | 0.040 | 0.093 |
| = | 3 | Poor peer relationships | 7 | 0.052 | 0.039 | 0.088 |
| = | 3 | Poor school attendance | 7 | 0.052 | 0.039 | 0.089 |
| = | 3 | Poor teacher-pupil relationship | 7 | 0.052 | 0.041 | 0.063 |
|  | 7 | Having an Education and Health Care Plan (EHCP) | 7 | 0.050 | 0.031 | 0.083 |
|  | 8 | Out-of-school discipline (e.g. suspension and expulsion) | 6 | 0.050 | 0.039 | 0.099 |
|  | 9 | Poor school climate (e.g. Ofsted weightings) | 6 | 0.049 | 0.031 | 0.063 |
|  | 10 | Special Educational Needs (SEN) | 7 | 0.049 | 0.032 | 0.085 |
|  | 11 | Poor quality or a lack of social-emotional learning programmes in pre-school | 7 | 0.047 | 0.014 | 0.061 |
| = | 12 | Home schooling (not by choice) | 7 | 0.046 | 0.032 | 0.083 |
| = | 12 | Negative experience of home schooling | 7 | 0.046 | 0.026 | 0.099 |
|  | 14 | Increased pressure/stress for teachers (e.g. insufficient pay/resources and poor leadership) | 6 | 0.042 | 0.007 | 0.072 |
|  | 15 | Language impairment in primary caregiver(s) | 7 | 0.039 | 0.013 | 0.072 |
|  | 16 | Poor educational attainment | 7 | 0.037 | 0.016 | 0.076 |
|  | 17 | Unemployment of the individual | 6 | 0.035 | 0.011 | 0.052 |
|  | 18 | Lack of imitation games with caregivers by age 1 | 5 | 0.032 | 0.016 | 0.050 |
|  | 19 | Serving in the military | 3 | 0.030 | 0.019 | 0.031 |
|  | 20 | Low education level of primary caregiver(s) | 7 | 0.029 | 0.012 | 0.057 |
|  | 21 | Child not read to daily by age 1 | 6 | 0.029 | 0.004 | 0.044 |
|  | 22 | School-level deprivation (e.g. proportion eligible for free school meals) | 6 | 0.020 | 0.008 | 0.058 |
|  | 23 | Participation in the Free-Lunch Program | 6 | 0.019 | 0.011 | 0.031 |
|  | 24 | School composition (e.g. size/headcount, gender proportions, ethnicity proportions) | 5 | 0.019 | 0.010 | 0.038 |
|  | 25 | Low education level of caregiver(s) (other than primary caregiver(s)) | 6 | 0.018 | 0.003 | 0.039 |
|  |  |  | **Range: 3 - 7** | | | |

Key: **CI** = confidence intervals

*Table S6: Ranked importance of risk factors within domain 4: Biomarkers*

| **Tied position** | **Rank within domain (out of 48)** | **Risk factor** | **Number of raters (N)** | **Mean score** | **Lower bound (CI)** | **Upper bound (CI)** |
| --- | --- | --- | --- | --- | --- | --- |
|  | 1 | Low intelligence quotient (IQ) | 3 | 0.077 | 0.052 | 0.171 |
|  | 2 | Genetic markers | 3 | 0.057 | 0.027 | 0.108 |
|  | 3 | Sex (biological) | 3 | 0.048 | 0.036 | 0.081 |
|  | 4 | Nutritional deficits | 2 | 0.044 | 0.037 | 0.051 |
|  | 5 | Neurotoxicity | 3 | 0.044 | 0.043 | 0.123 |
|  | 6 | Neuronal atrophy | 2 | 0.043 | 0.043 | 0.043 |
|  | 7 | Increased neurodegeneration | 3 | 0.042 | 0.038 | 0.147 |
|  | 8 | Reduced neuroplasticity | 2 | 0.042 | 0.041 | 0.042 |
|  | 9 | Low levels of dopamine | 2 | 0.041 | 0.037 | 0.046 |
|  | 10 | Low levels of nutrients | 2 | 0.041 | 0.034 | 0.048 |
|  | 11 | Low levels of serotonin | 2 | 0.041 | 0.037 | 0.045 |
|  | 12 | Reduced neurogenesis | 2 | 0.041 | 0.039 | 0.042 |
|  | 13 | HPA axis and glucocorticoid receptor resistance | 1 | 0.039 | 0.039 | 0.039 |
|  | 14 | Low levels of folate | 1 | 0.037 | 0.037 | 0.037 |
|  | 15 | Reduced levels of BDNF (i.e. neurotrophic factor) | 1 | 0.036 | 0.036 | 0.036 |
|  | 16 | Reduced grey matter in hippocampus | 2 | 0.036 | 0.030 | 0.042 |
|  | 17 | Raised levels of glucocorticoids/cortisol | 2 | 0.035 | 0.030 | 0.039 |
|  | 18 | High levels of cytokine proteins (e.g. interleukin-1 [IL-1]; interleukin-6 [IL-6]) | 1 | 0.035 | 0.035 | 0.035 |
|  | 19 | Birth weight | 3 | 0.034 | 0.022 | 0.109 |
|  | 20 | Raised C-reactive protein (CRP) | 2 | 0.033 | 0.029 | 0.037 |
|  | 21 | Low levels of vitamin D | 2 | 0.032 | 0.028 | 0.037 |
|  | 22 | Decreased neuronal branching | 2 | 0.032 | 0.025 | 0.039 |
|  | 23 | Low levels of melatonin | 1 | 0.031 | 0.031 | 0.031 |
|  | 24 | Low serum ferritin | 2 | 0.031 | 0.028 | 0.034 |
|  | 25 | Changes in prefrontal cortex (e.g. reduced total volume; low myelination; low white matter; increased middle inferior and ventral regions and superior/dorsal regions) | 2 | 0.029 | 0.013 | 0.045 |
| = | 26 | Age of parents at time of giving birth | 3 | 0.028 | 0.007 | 0.087 |
| = | 26 | High intelligence quotient (IQ) | 3 | 0.028 | 0.001 | 0.142 |
| = | 28 | Epigenetic marker - DNA methylation | 1 | 0.026 | 0.026 | 0.026 |
| = | 28 | Epigenetic marker - non-coding ribonucleic acids | 1 | 0.026 | 0.026 | 0.026 |
|  | 28 | Gut microbiome (reduced diversity and reduced populations of certain bacteria) | 1 | 0.026 | 0.026 | 0.026 |
|  | 31 | Epigenetic marker - chromatin regulation | 1 | 0.025 | 0.025 | 0.025 |
|  | 32 | Chronic inflammation | 1 | 0.024 | 0.024 | 0.024 |
|  | 33 | Changes in amygdala (e.g. reduced volume) | 2 | 0.024 | 0.017 | 0.031 |
|  | 34 | Birth length | 2 | 0.014 | 0.005 | 0.022 |
|  |  |  | **Range: 1 - 3** | | | |

Key: **CI** = confidence intervals

*Table S7: Ranked importance of risk factors within domain 5: Physical Health*

| **Tied position** | **Rank within domain (out of 52)** | **Risk factor** | **Number of raters (N)** | **Mean score** | **Lower bound (CI)** | **Upper bound (CI)** |
| --- | --- | --- | --- | --- | --- | --- |
|  | 1 | Traumatic brain injury | 6 | 0.047 | 0.022 | 0.092 |
|  | 2 | Primary caregiver(s) chronic (long lasting) illness | 6 | 0.043 | 0.022 | 0.086 |
|  | 3 | Severe health condition | 6 | 0.042 | 0.023 | 0.068 |
|  | 4 | Severe illness in family | 6 | 0.040 | 0.022 | 0.092 |
|  | 5 | Sleep disorder | 6 | 0.037 | 0.023 | 0.091 |
|  | 6 | Physical disability | 6 | 0.036 | 0.017 | 0.043 |
|  | 7 | Global developmental delay | 6 | 0.034 | 0.020 | 0.045 |
|  | 8 | Prolonged duration of a physical health condition | 5 | 0.034 | 0.014 | 0.065 |
|  | 9 | Irritable Bowel Syndrome (IBS) | 5 | 0.033 | 0.017 | 0.070 |
|  | 10 | Lack of response to treatment for a physical health condition | 4 | 0.030 | 0.020 | 0.042 |
|  | 11 | Diabetes | 6 | 0.028 | 0.010 | 0.041 |
|  | 12 | Hearing impairment causing disability (e.g. deafness) | 6 | 0.028 | 0.021 | 0.046 |
|  | 13 | Autoimmune disorders (e.g. rheumatoid arthritis) | 6 | 0.027 | 0.014 | 0.034 |
|  | 14 | Hypoxia (at birth) | 4 | 0.027 | 0.006 | 0.047 |
|  | 15 | Maternal obesity/overweight during pregnancy | 2 | 0.026 | 0.020 | 0.032 |
|  | 16 | Visual impairment causing disability (e.g. blindness/partial sightedness) | 5 | 0.025 | 0.017 | 0.073 |
|  | 17 | Thyroid disease | 5 | 0.024 | 0.015 | 0.043 |
|  | 18 | Congenital malformations | 5 | 0.023 | 0.011 | 0.046 |
|  | 19 | 5-min Apgar score <7 | 6 | 0.022 | 0.001 | 0.034 |
|  | 20 | Asthma | 6 | 0.022 | 0.012 | 0.040 |
|  | 21 | Anaemia | 6 | 0.021 | 0.016 | 0.024 |
|  | 22 | Premature birth | 6 | 0.021 | 0.003 | 0.041 |
| = | 23 | Low diversity and composition of gut microbiota | 3 | 0.021 | 0.021 | 0.038 |
| = | 23 | Repeated infections | 5 | 0.021 | 0.012 | 0.029 |
|  | 25 | Pre-eclampsia | 4 | 0.021 | 0.002 | 0.021 |
|  | 26 | Inflammatory diseases (e.g. Lyme disease) | 5 | 0.021 | 0.014 | 0.033 |
| = | 27 | Maternal hypertension during pregnancy | 1 | 0.021 | 0.021 | 0.021 |
| = | 27 | Maternal infection requiring hospitalisation during pregnancy | 1 | 0.021 | 0.021 | 0.021 |
| = | 27 | Maternal diabetes during pregnancy | 1 | 0.021 | 0.021 | 0.021 |
| = | 27 | Chronic (long lasting) infection (e.g. Lyme disease, periodontal disease) | 5 | 0.021 | 0.006 | 0.034 |
| = | 31 | Low serum vitamin D | 5 | 0.021 | 0.001 | 0.025 |
| = | 31 | Maternal auto-immune disease during pregnancy | 1 | 0.021 | 0.021 | 0.021 |
|  | 33 | Perinatal infections (e.g. cytomegalovirus) | 6 | 0.020 | 0.002 | 0.039 |
|  | 34 | Family history of autoimmune disease | 6 | 0.020 | 0.000 | 0.023 |
|  | 35 | Chronic (long lasting) gastric ill-health (e.g. inflammatory bowel disease) | 5 | 0.020 | 0.009 | 0.039 |
|  | 36 | Obstetric complications (other) | 4 | 0.019 | 0.001 | 0.032 |
|  | 37 | Disease history | 3 | 0.018 | 0.011 | 0.027 |
|  | 38 | Dental caries (tooth decay) | 6 | 0.017 | 0.003 | 0.030 |
|  | 39 | Chronic (long lasting) reflux or indigestion | 5 | 0.017 | 0.001 | 0.033 |
|  | 40 | Allergies (e.g. non-lgE-mediated food allergies, pollen allergies) | 6 | 0.017 | 0.000 | 0.021 |
|  | 41 | Family history of psoriasis | 4 | 0.016 | 0.011 | 0.023 |
|  | 42 | Obesity/overweight | 4 | 0.016 | 0.011 | 0.023 |
|  | 43 | Body Mass Index (BMI) | 5 | 0.016 | 0.009 | 0.055 |
|  | 44 | Eczema | 6 | 0.015 | 0.001 | 0.033 |
|  | 45 | Long term antibiotic use | 4 | 0.015 | 0.008 | 0.039 |
|  | 46 | Family history of Type 1 diabetes | 6 | 0.014 | 0.000 | 0.021 |
|  | 47 | Family history of rheumatoid arthritis | 4 | 0.013 | 0.001 | 0.023 |
|  | 48 | Maternal self-rated health (prior to pregnancy) | 4 | 0.013 | 0.000 | 0.036 |
|  | 49 | Dental erosion | 4 | 0.009 | 0.000 | 0.030 |
|  | 50 | Polyhydramnios | 3 | 0.005 | 0.000 | 0.020 |
|  | 51 | Ruptured membranes (at birth) | 4 | 0.003 | 0.000 | 0.018 |
|  |  |  | **Range: 1 - 6** | | | |

Key: **CI** = confidence intervals

*Table S8: Ranked importance of risk factors within domain 6: Psychological and Mental Health*

| **Tied position** | **Rank within domain (out of 26)** | **Risk factor** | **Number of raters (N)** | **Mean score** | **Lower bound (CI)** | **Upper bound (CI)** |
| --- | --- | --- | --- | --- | --- | --- |
|  | 1 | Family history of severe mental illness (e.g. psychosis) | 14 | 0.050 | 0.034 | 0.071 |
|  | 2 | Primary caregiver(s) mental health problems | 14 | 0.046 | 0.038 | 0.072 |
|  | 3 | Psychiatric history | 14 | 0.045 | 0.037 | 0.075 |
|  | 4 | Family history of psychiatric disorders | 14 | 0.043 | 0.034 | 0.074 |
|  | 5 | Social-communication problems | 14 | 0.043 | 0.030 | 0.063 |
|  | 6 | Neuro-developmental conditions (e.g. autism) | 13 | 0.042 | 0.036 | 0.069 |
|  | 7 | Increased panic attacks | 13 | 0.042 | 0.032 | 0.061 |
|  | 8 | Anxiety (as a predictor of further mental health problems) | 14 | 0.040 | 0.031 | 0.069 |
| = | 9 | High levels of perceived stress | 13 | 0.040 | 0.034 | 0.067 |
| = | 9 | Ineffective coping strategies | 13 | 0.040 | 0.027 | 0.057 |
|  | 11 | Maternal psychosis (perinatal or pre-natal) | 12 | 0.040 | 0.023 | 0.054 |
|  | 12 | Lack of psychological resilience | 14 | 0.040 | 0.026 | 0.067 |
|  | 13 | Attention (as a predictor of further mental health problems) | 13 | 0.040 | 0.027 | 0.061 |
|  | 14 | Intellectual disability | 12 | 0.040 | 0.022 | 0.052 |
|  | 15 | Increased anxiety arousal | 13 | 0.039 | 0.010 | 0.046 |
|  | 16 | Dissociation during traumatic experience | 12 | 0.039 | 0.026 | 0.051 |
|  | 17 | Acute stress symptoms (anxiety, avoidance or depression - as a predictor of further mental health problems) | 14 | 0.039 | 0.014 | 0.069 |
|  | 18 | Acute stress disorder (as a predictor of further mental health problems) | 14 | 0.039 | 0.020 | 0.066 |
|  | 19 | Excessive rumination | 13 | 0.039 | 0.022 | 0.049 |
|  | 20 | Poor concentration | 13 | 0.039 | 0.018 | 0.046 |
|  | 21 | Negative attitudes to mental health problems within society an individual lives in | 14 | 0.038 | 0.000 | 0.070 |
|  | 22 | Maternal depression during pregnancy | 12 | 0.038 | 0.012 | 0.044 |
|  | 23 | Poor problem-solving abilities | 13 | 0.037 | 0.017 | 0.048 |
|  | 24 | Emotional reactivity | 14 | 0.036 | 0.007 | 0.062 |
|  | 25 | Poor visuospatial functioning | 10 | 0.036 | 0.012 | 0.042 |
|  | 26 | Problems with memory | 13 | 0.035 | 0.019 | 0.045 |
|  |  |  | **Range: 10 - 14** | | | |

Key: **CI** = confidence intervals

*Table S9: Ranked importance of risk factors within domain 7: Patterns of Service Use*

| **Tied position** | **Rank within domain (out of 7)** | **Risk factor** | **Number of raters (N)** | **Mean score** | **Lower bound (CI)** | **Upper bound (CI)** |
| --- | --- | --- | --- | --- | --- | --- |
|  | 1 | Child protection record | 6 | 0.203 | 0.158 | 0.273 |
|  | 2 | Investigations by multiple services suggestive of suffering from medically unexplained symptoms | 7 | 0.186 | 0.149 | 0.248 |
|  | 3 | Failure to attend three or more planned health or social care appointments | 7 | 0.168 | 0.115 | 0.230 |
|  | 4 | Three or more presentations to emergency services within a year | 6 | 0.158 | 0.085 | 0.387 |
|  | 5 | Repeat hospitalisation | 6 | 0.144 | 0.109 | 0.288 |
|  | 6 | Missed ante-natal visits | 6 | 0.120 | 0.024 | 0.247 |
|  | 7 | Primary caregiver(s) non-attendance at baby groups | 7 | 0.023 | 0.000 | 0.104 |
|  |  |  | **Range: 6 - 7** | | | |

Key: **CI** = confidence intervals

*Table S10: Ranked importance of risk factors within domain 8: Factors Identified to be Particularly Relevant to Under-Served Populations*

| **Tied position** | **Rank within domain (out of 102)** | **Risk factor** | **Number of raters (N)** | **Mean score** | **Lower bound (CI)** | **Upper bound (CI)** |
| --- | --- | --- | --- | --- | --- | --- |
|  | 1 | Being a looked after child (LAC) | 5 | 0.016 | 0.011 | 0.029 |
|  | 2 | Child in need (CIN) status | 5 | 0.016 | 0.011 | 0.021 |
|  | 3 | Torture | 5 | 0.015 | 0.012 | 0.019 |
|  | 4 | Separation from family (e.g. out-of-home care) | 6 | 0.015 | 0.012 | 0.027 |
|  | 5 | Child protection record | 6 | 0.015 | 0.012 | 0.022 |
|  | 6 | Heavy alcohol use | 6 | 0.014 | 0.012 | 0.023 |
|  | 7 | Victim of organised crime (e.g. commercial sexual exploitation or via county lines) | 5 | 0.014 | 0.012 | 0.021 |
|  | 8 | Imprisonment | 5 | 0.014 | 0.012 | 0.029 |
|  | 9 | Homelessness (young person has left home) | 6 | 0.014 | 0.012 | 0.025 |
|  | 10 | Primary caregiver(s) mental health problems | 6 | 0.014 | 0.011 | 0.028 |
|  | 11 | Victim or witness of violent crime | 6 | 0.013 | 0.007 | 0.016 |
|  | 12 | Experience of bereavement during a traumatic event¬ | 6 | 0.013 | 0.010 | 0.018 |
|  | 13 | Victim of structural/systemic racism | 6 | 0.013 | 0.003 | 0.017 |
|  | 14 | Being an asylum-seeking family | 6 | 0.013 | 0.009 | 0.018 |
|  | 15 | Homelessness | 6 | 0.013 | 0.011 | 0.018 |
|  | 16 | Household mental illness | 6 | 0.013 | 0.012 | 0.023 |
|  | 17 | War/conflict | 6 | 0.013 | 0.011 | 0.016 |
| = | 18 | Gangland crime | 6 | 0.013 | 0.004 | 0.016 |
| = | 18 | Family experience of social exclusion, discrimination and harassment associated with ethnicity | 6 | 0.013 | 0.003 | 0.017 |
|  | 20 | Witnessing injury/death during a traumatic event | 6 | 0.013 | 0.011 | 0.016 |
|  | 21 | Neuro-developmental conditions (e.g. autism) | 6 | 0.013 | 0.008 | 0.017 |
|  | 22 | Experience of racism and discrimination | 6 | 0.013 | 0.009 | 0.020 |
|  | 23 | Disabled child/young person experiencing infantilisation | 2 | 0.013 | 0.012 | 0.013 |
|  | 24 | Out-of-school discipline (e.g. suspension and expulsion) | 6 | 0.013 | 0.012 | 0.024 |
|  | 25 | Being an unaccompanied asylum seeker | 6 | 0.012 | 0.012 | 0.021 |
|  | 26 | Famine | 5 | 0.012 | 0.004 | 0.016 |
| = | 27 | Household drug abuse | 6 | 0.012 | 0.011 | 0.016 |
| = | 27 | Household member involvement in criminal justice system | 6 | 0.012 | 0.012 | 0.013 |
|  | 29 | Being a young carer | 6 | 0.012 | 0.005 | 0.017 |
|  | 30 | Radicalisation | 6 | 0.012 | 0.002 | 0.016 |
|  | 31 | Organised crime affiliation | 5 | 0.012 | 0.010 | 0.013 |
|  | 32 | Household alcohol abuse | 6 | 0.012 | 0.010 | 0.016 |
|  | 33 | Involvement in criminal justice system | 6 | 0.012 | 0.011 | 0.027 |
|  | 34 | Witness of community violence | 6 | 0.012 | 0.007 | 0.013 |
|  | 35 | School exclusions | 6 | 0.012 | 0.008 | 0.024 |
|  | 36 | Family has low trust in services | 6 | 0.012 | 0.009 | 0.017 |
|  | 37 | Household criminality | 6 | 0.012 | 0.011 | 0.015 |
|  | 38 | Imprisonment of household member | 5 | 0.012 | 0.006 | 0.012 |
|  | 39 | Non-prescription drug use | 6 | 0.012 | 0.009 | 0.014 |
|  | 40 | Poor school attendance | 6 | 0.012 | 0.009 | 0.026 |
|  | 41 | Maternal substance abuse during pregnancy | 6 | 0.012 | 0.005 | 0.015 |
|  | 42 | Poor school climate (e.g. Ofsted weightings) | 6 | 0.011 | 0.009 | 0.015 |
|  | 43 | Being an adoptee | 5 | 0.011 | 0.005 | 0.012 |
|  | 44 | Cultural racism | 6 | 0.011 | 0.003 | 0.018 |
|  | 45 | Prescription drug abuse | 6 | 0.011 | 0.008 | 0.017 |
|  | 46 | Having an Education and Health Care Plan (EHCP) | 5 | 0.011 | 0.009 | 0.026 |
|  | 47 | Failure to attend three or more planned health or social care appointments | 6 | 0.011 | 0.008 | 0.024 |
|  | 48 | Maternal use of psychotropics during pregnancy | 5 | 0.011 | 0.004 | 0.016 |
|  | 49 | Maternal alcohol use during pregnancy | 6 | 0.011 | 0.003 | 0.015 |
|  | 50 | Ethnic minority in low ethnic density area | 6 | 0.011 | 0.003 | 0.016 |
|  | 51 | Special Educational Needs (SEN) | 6 | 0.011 | 0.006 | 0.012 |
|  | 52 | Quality of school climate (e.g. relating to school connectedness, feelings of safety in school, perception of school, adult-student relationships, morale) | 6 | 0.011 | 0.006 | 0.016 |
|  | 53 | Poverty | 6 | 0.011 | 0.004 | 0.013 |
|  | 54 | Poor educational attainment | 6 | 0.011 | 0.008 | 0.014 |
|  | 55 | Intellectual disability | 6 | 0.010 | 0.003 | 0.018 |
| = | 56 | Poor quality or a lack of social-emotional learning programmes in pre-school | 5 | 0.010 | 0.004 | 0.015 |
| = | 56 | Maternal depression during pregnancy | 5 | 0.010 | 0.004 | 0.012 |
|  | 58 | Physical disability | 6 | 0.010 | 0.005 | 0.012 |
|  | 59 | Maternal psychosis (perinatal or pre-natal) | 5 | 0.010 | 0.006 | 0.015 |
|  | 60 | Negative previous experiences of services | 6 | 0.010 | 0.009 | 0.020 |
|  | 61 | Being a young parent | 6 | 0.010 | 0.003 | 0.013 |
|  | 62 | Household overcrowding | 6 | 0.010 | 0.003 | 0.012 |
|  | 63 | Family financial problems | 6 | 0.010 | 0.005 | 0.012 |
|  | 64 | Area deprivation (area code) | 6 | 0.010 | 0.004 | 0.016 |
|  | 65 | Food poverty | 6 | 0.010 | 0.003 | 0.012 |
|  | 66 | Minority gender identity | 4 | 0.010 | 0.002 | 0.017 |
|  | 67 | Racial bias in obstetric care | 5 | 0.010 | 0.001 | 0.014 |
|  | 68 | School-level deprivation (e.g. proportion eligible for free school meals) | 6 | 0.009 | 0.002 | 0.011 |
|  | 69 | Personal direct experience of natural disaster | 6 | 0.009 | 0.005 | 0.012 |
|  | 70 | Living in an area with high crime levels | 6 | 0.009 | 0.001 | 0.013 |
|  | 71 | Increased pressure/stress for teachers (e.g. insufficient pay/resources and poor leadership) | 6 | 0.009 | 0.002 | 0.015 |
|  | 72 | Language impairment in primary caregiver(s) | 6 | 0.009 | 0.001 | 0.015 |
|  | 73 | Unemployment of the individual | 6 | 0.008 | 0.005 | 0.013 |
|  | 74 | Trapped during earthquake | 5 | 0.008 | 0.006 | 0.014 |
|  | 75 | Minority sexuality | 5 | 0.008 | 0.002 | 0.015 |
|  | 76 | Primary caregiver(s) parenting styles (strict/rigid/conventional) | 6 | 0.008 | 0.001 | 0.021 |
|  | 77 | Hearing impairment causing disability (e.g. deafness) | 6 | 0.008 | 0.002 | 0.013 |
|  | 78 | Primary caregiver(s) unemployment | 6 | 0.008 | 0.001 | 0.010 |
|  | 79 | Social-communication problems | 6 | 0.007 | 0.005 | 0.012 |
|  | 80 | Air pollution | 6 | 0.007 | 0.002 | 0.014 |
|  | 81 | Participation in the Free-Lunch Program | 6 | 0.007 | 0.003 | 0.010 |
|  | 82 | Low education level of primary caregiver(s) | 6 | 0.007 | 0.004 | 0.011 |
|  | 83 | School composition (e.g. size/headcount, gender proportions, ethnicity proportions) | 4 | 0.007 | 0.002 | 0.011 |
|  | 84 | Multi-generational families within the same home | 6 | 0.006 | 0.000 | 0.008 |
|  | 85 | Migration from another country | 6 | 0.006 | 0.001 | 0.011 |
|  | 86 | Visual impairment causing disability (e.g. blindness/partial sightedness) | 5 | 0.006 | 0.003 | 0.012 |
| = | 87 | Serving in the military | 3 | 0.006 | 0.006 | 0.012 |
| = | 87 | Missed ante-natal visits | 5 | 0.006 | 0.002 | 0.011 |
|  | 89 | English as an additional language | 6 | 0.006 | 0.000 | 0.015 |
|  | 90 | Nitrous oxide use | 2 | 0.006 | 0.003 | 0.009 |
|  | 91 | Digital exclusion | 4 | 0.006 | 0.004 | 0.013 |
|  | 92 | Low socioeconomic status | 6 | 0.006 | 0.002 | 0.013 |
|  | 93 | Remoteness of living | 6 | 0.006 | 0.000 | 0.013 |
|  | 94 | Low education level of caregiver(s) (other than primary caregiver(s)) | 6 | 0.005 | 0.001 | 0.011 |
|  | 95 | Urbanicity | 5 | 0.005 | 0.001 | 0.011 |
|  | 96 | Ethnicity | 5 | 0.005 | 0.002 | 0.010 |
|  | 97 | Climate change | 5 | 0.005 | 0.002 | 0.010 |
|  | 98 | Belonging to a religious minority | 6 | 0.005 | 0.000 | 0.011 |
|  | 99 | Belonging to a traveller community | 5 | 0.004 | 0.001 | 0.010 |
|  | 100 | Religiosity | 5 | 0.003 | 0.000 | 0.007 |
|  | 101 | Religiosity of caregiver(s) | 6 | 0.001 | 0.000 | 0.011 |
|  | 102 | Being a second generation immigrant | 6 | 0.001 | 0.000 | 0.011 |
|  |  |  | **Range: 2 - 6** | | | |

Key: **CI** = confidence intervals

**¬** Of note, the risk factor ‘Experience of bereavement during a traumatic event’ was accidentally missed from the main framework (domains 1-7) and only features in domain 8. As such, there are actually 288 unique risk factors in the whole framework but only 287 in the main framework (hence, 287 was used as the reported total).

####

#### **Reference list for developing the theoretical framework of risk factors**

Boyd, M., Kisely, S., Najman, J., & Mills, R. (2019). Child maltreatment and attentional problems: A longitudinal birth cohort study. Child Abuse and Neglect, 98(August), 104170. <https://doi.org/10.1016/j.chiabu.2019.104170>

Cant, R. L., O’Donnell, M., Sims, S., & Harries, M. (2019). Overcrowded housing: One of a constellation of vulnerabilities for child sexual abuse. Child Abuse and Neglect, 93(May), 239– 248. <https://doi.org/10.1016/j.chiabu.2019.05.010>

Carvalho, A. F., Solmi, M., Sanches, M., Machado, M. O., Stubbs, B., Ajnakina, O., Sherman, C., Sun, Y. R., Liu, C. S., Brunoni, A. R., Pigato, G., Fernandes, B. S., Bortolato, B., Husain, M. I., Dragioti, E., Firth, J., Cosco, T. D., Maes, M., Berk, M., ... Herrmann, N. (2020). Evidence-based umbrella review of 162 peripheral biomarkers for major mental disorders. Translational Psychiatry, 10(1). <https://doi.org/10.1038/s41398-020-0835-5>

Davies, C., Segre, G., Estradé, A., Radua, J., De Micheli, A., Provenzani, U., Oliver, D., Salazar de Pablo, G., Ramella-Cravaro, V., Besozzi, M., Dazzan, P., Miele, M., Caputo, G., Spallarossa, C., Crossland, G., Ilyas, A., Spada, G., Politi, P., Murray, R. M., ... Fusar-Poli, P. (2020). Prenatal and perinatal risk and protective factors for psychosis: a systematic review and meta-analysis. The Lancet Psychiatry, 7(5), 399–410. <https://doi.org/10.1016/S2215-0366(20)30057-2>

Deighton, J., Humphrey, N., Belsky, J., Boehnke, J., Vostanis, P., & Patalay, P. (2018). Longitudinal pathways between mental health difficulties and academic performance during middle childhood and early adolescence. British Journal of Developmental Psychology, 36(1), 110–126. <https://doi.org/10.1111/bjdp.12218>

Deighton, J., Lereya, S. T., Casey, P., Patalay, P., Humphrey, N., & Wolpert, M. (2019). Prevalence of mental health problems in schools: Poverty and other risk factors among 28 000 adolescents in England. British Journal of Psychiatry, 215(3), 565–567. <https://doi.org/10.1192/bjp.2019.19>

Felitti, V. J., Anda, R. F., Nordenberg, D., Williamson, D. F., Spitz, A. M., Edwards, V., & Marks, J. S. (1998). Relationship of childhood abuse and household dysfunction to many of the leading causes of death in adults: The Adverse Childhood Experiences (ACE) Study. American journal of preventive medicine, 14(4), 245-258.

Fullana, M. A., Tortella-Feliu, M., Fernández de la Cruz, L., Chamorro, J., Pérez-Vigil, A., Ioannidis, J., Solanes, A., Guardiola, M., Almodóvar, C., Miranda-Olivos, R., Ramella-Cravaro, V., Vilar, A., Reichenberg, A., Mataix-Cols, D., Vieta, E., Fusar-Poli, P., Fatjó-Vilas, M., & Radua, J. (2020). Risk and protective factors for anxiety and obsessive-compulsive disorders: an umbrella review of systematic reviews and meta-analyses. Psychological Assessment, 50, 1300–1315. <https://doi.org/10.1017/S0033291719001247>

Fusar-Poli, P., Tantardini, M., De Simone, S., Ramella-Cravaro, V., Oliver, D., Kingdon, J., Kotlicka- Antczak, M., Valmaggia, L., Lee, J., Millan, M. J., Galderisi, S., Balottin, U., Ricca, V., & McGuire, P. (2017). Deconstructing vulnerability for psychosis: Meta-analysis of environmental risk factors for psychosis in subjects at ultra high-risk. European Psychiatry, 40, 65–75. <https://doi.org/10.1016/j.eurpsy.2016.09.003>

Hovenkamp-Hermelink, J. H. M., Jeronimus, B. F., Myroniuk, S., Riese, H., & Schoevers, R. A. (2021). Predictors of persistence of anxiety disorders across the lifespan: a systematic review. The Lancet Psychiatry, 0366(20), 1–16. <https://doi.org/10.1016/s2215-0366(20)30433-8>

Kim, J. H., Kim, J. Y., Lee, J., Jeong, G. H., Lee, E., Lee, S., Lee, K. H., Kronbichler, A., Stubbs, B., Solmi, M., Koyanagi, A., Hong, S. H., Dragioti, E., Jacob, L., Brunoni, A. R., Carvalho, A. F., Radua, J., Thompson, T., Smith, L., ... Fusar-Poli, P. (2020). Environmental risk factors, protective factors, and peripheral biomarkers for ADHD: an umbrella review. The Lancet Psychiatry, 7(11), 955– 970. <https://doi.org/10.1016/S2215-0366(20)30312-6>

Lereya, S. T., Patel, M., dos Santos, J. P. G. A., & Deighton, J. (2019). Mental Health Difficulties, Attainment & Attendance: A Cross-sectional Study. Eur Child Adolesc Psychiatry, 28(8), 1147–1152. <https://doi.org/10.1007/s00787-018-01273-6>

National Academies of Sciences Engineering and Medicine. (2019). Fostering Healthy Mental, Emotional, and Behavioral Development in Children and Youth: A National Agenda. National Academies Press. <https://doi.org/10.17226/25201>

National Research Council and Institute of Medicine. (2009). Preventing mental, emotional, and behavioral disorders among young people: Progress and possibilities. National Academies Press. <https://doi.org/10.17226/12480>

Oliver, D., Reilly, T. J., Baccaredda Boy, O., Petros, N., Davies, C., Borgwardt, S., McGuire, P., & Fusar- Poli, P. (2020). What Causes the Onset of Psychosis in Individuals at Clinical High Risk? A Meta- analysis of Risk and Protective Factors. Schizophrenia Bulletin, 46(1), 110–120. <https://doi.org/10.1093/schbul/sbz039>

Patalay, P., O’Neill, E., Deighton, J., & Fink, E. (2020). School characteristics and children’s mental health: A linked survey-administrative data study. Preventive Medicine, 141(February), 106292. <https://doi.org/10.1016/j.ypmed.2020.106292>

Radua, J., Ramella-Cravaro, V., Ioannidis, J. P. A., Reichenberg, A., Phiphopthatsanee, N., Amir, T., Yenn Thoo, H., Oliver, D., Davies, C., Morgan, C., McGuire, P., Murray, R. M., & Fusar-Poli, P. (2018). What causes psychosis? An umbrella review of risk and protective factors. World Psychiatry, 17(1), 49–66. <https://doi.org/10.1002/wps.20490>

Rutigliano, G., Rocchetti, M., Paloyelis, Y., Gilleen, J., Sardella, A., Cappucciati, M., Palombini, E., Dell’Osso, L., Caverzasi, E., Politi, P., McGuire, P., & Fusar-Poli, P. (2016). Peripheral oxytocin and vasopressin: Biomarkers of psychiatric disorders? A comprehensive systematic review and preliminary meta-analysis. Psychiatry Research, 241, 207–220. <https://doi.org/10.1016/j.psychres.2016.04.117>

Solmi, M., Radua, J., Stubbs, B., Ricca, V., Moretti, D., Busatta, D., Carvalho, A. F., Dragioti, E., Favaro, A., Monteleone, A. M., Shin, J. Il, Fusar-Poli, P., & Castellini, G. (2020). Risk factors for eating disorders: an umbrella review of published meta-analyses. Brazilian Journal of Psychiatry, 00(00), 1–10. <https://doi.org/10.1590/1516-4446-2020-1099>

The Foundation for Young People’s Mental Health. (2020). A new approach to understanding and treating depression in young people: Stakeholder consultation document. <https://www.ypmh.org/changingminds/>

Tortella-Feliu, M., Fullana, M. A., Pérez-Vigil, A., Torres, X., Chamorro, J., Littarelli, S. A., Solanes, A., Ramella-Cravaro, V., Vilar, A., González-Parra, J. A., Andero, R., Reichenberg, P. A., Mataix-Cols, P. D., Vieta, E., Fusar-Poli, P., Ioannidis, P. J. P. A., Stein, P. M. B., Radua, J., & Fernández de la Cruz, L. (2019). Risk factors for posttraumatic stress disorder: An umbrella review of systematic reviews and meta-analyses. Neuroscience and Biobehavioral Reviews, 107(July), 154–165. <https://doi.org/10.1016/j.neubiorev.2019.09.013>

Zimmermann, M., Chong, A. K., Vechiu, C., & Papa, A. (2020). Modifiable risk and protective factors for anxiety disorders among adults: A systematic review. Psychiatry Research, 285(November 2019), 112705. <https://doi.org/10.1016/j.psychres.2019.112705>
