# Supplementary material for: Development of a risk factor framework to inform machine learning prediction of young people’s mental health problems: a Delphi study
Source: JAMIA Open. 2025 Dec 23;8(6):ooaf166. doi: 10.1093/jamiaopen/ooaf166 (PMC12726920; doi:10.1093/jamiaopen/ooaf166)
Supplement: ooaf166_Supplementary_Data [file ooaf166_supplementary_data.zip › Supplementary File 2 - Final Framework of Risk Factors.pdf]

Domain 1: Social and Environmental			
Rank within Domain	Risk Factor	Level	Number of Raters (N)
1	Being a looked after child (LAC)	Individual	14
2	Torture	Individual	13
3	Sexual abuse	Individual	16
4	Lack of supportive relationship(s) with a trusted adult	Society	15
=5	Poor primary caregiver-caregiver relationship	Family/Caregiver	16
=5	Cumulative traumatic events	Individual	16
=7	Domestic violence	Family/Caregiver	16
=7	Trauma severity	Individual	14
9	Emotional, psychological or verbal abuse	Individual	16
10	Emotional neglect	Individual	16
11	Victim of organised crime (e.g. commercial sexual exploitation or via county lines)	Individual	13
=12	Household drug abuse	Family/Caregiver	16
=12	Poor primary caregiver-child relationship	Individual	16
14	Physical abuse	Individual	16
15	Household mental illness	Family/Caregiver	16
16	Family conflict or discord	Family/Caregiver	16
17	Death of primary caregiver(s)	Family/Caregiver	16
18	Caregiver negative attitude to mental health problems	Family/Caregiver	16
19	Separation from family (e.g. out-of-home care)	Individual	15
=20	Homelessness	Family/Caregiver	16
=20	Chronic psychological stress	Individual	16
22	Household alcohol abuse	Family/Caregiver	16
23	Family experience of social exclusion, discrimination and harassment associated with ethnicity	Family/Caregiver	14
24	Victim or witness of violent crime	Individual	13
25	Social isolation	Individual	16
26	Physical neglect	Individual	16
27	Witnessing injury/death during a traumatic event	Individual	14
28	Being an unaccompanied asylum seeker	Individual	15
=29	Being an asylum-seeking family	Family/Caregiver	14
=29	Victim of bullying	Individual	16
=31	Experience of racism and discrimination	Individual	15
=31	Child in need (CIN) status	Individual	15
33	Maternal substance abuse during pregnancy	Individual	14
34	Imprisonment	Individual	14
35	Experience of past traumatic event	Individual	16
36	Frequent fear of family member	Individual	16
=37	Involvement in criminal justice system	Individual	15
=37	Homelessness (young person has left home)	Individual	15
39	War/conflict	Society	13
40	Victim of structural/systemic racism	Society	13

41	Witness of community violence	Individual	13
42	Household criminality	Family/Caregiver	16
43	Area deprivation (area code)	Society	15
44	Injury during a traumatic event	Individual	14
45	Household member involvement in criminal justice system	Family/Caregiver	16
46	Disabled child/young person experiencing infantilisation	Individual	10
47	Maternal lead exposure during pregnancy	Individual	8
48	Maternal use of psychotropics during pregnancy	Individual	11
49	Imprisonment of household member	Family/Caregiver	14
50	Maternal alcohol use during pregnancy	Individual	15
51	Poor peer relationships	Individual	16
52	Cultural racism	Society	14
53	Organised crime affiliation	Individual	12
54	Death of a close friend	Individual	16
55	Trapped during earthquake	Individual	11
56	Family has low trust in services	Family/Caregiver	15
57	Famine	Society	12
58	Family financial problems	Family/Caregiver	15
59	Low social support for family	Family/Caregiver	16
60	Being an adoptee	Individual	15
61	Gangland crime	Society	13
62	Being a young carer	Individual	16
63	Frequently moving homes	Family/Caregiver	15
=64	Ethnic minority in low ethnic density area	Society	13
=64	Experiencing financial abuse	Individual	13
66	Personal direct experience of natural disaster	Individual	16
67	Household overcrowding	Family/Caregiver	14
68	Perceived pressure to be thin	Individual	16
69	Poverty	Family/Caregiver	16
70	Minority gender identity	Individual	11
71	Food poverty	Family/Caregiver	13
72	Poor online peer relationships	Individual	16
73	Minority sexuality	Individual	12
74	Death of close relative (excluding primary caregiver(s))	Family/Caregiver	16
75	Being a young parent	Individual	16
76	Radicalisation	Society	12
77	English as an additional language	Society	13
78	Racial bias in obstetric care	Society	11
79	Maternal smoking during pregnancy	Individual	12
80	Low socioeconomic status family	Family/Caregiver	16
81	Primary caregiver(s) parenting styles (strict/rigid/conventional)	Family/Caregiver	16
82	Living in an area with high crime levels	Society	13
83	Migration from another country	Family/Caregiver	14
84	Remoteness of living	Society	12

85	Belonging to a traveller community	Family/Caregiver	14
86	Negative previous experiences of services	Individual	16
87	Belonging to a religious minority	Family/Caregiver	14
88	Air pollution	Society	12
89	Blended or complex family structure	Family/Caregiver	16
90	Primary caregiver(s) unemployment	Family/Caregiver	16
91	Digital exclusion	Individual	11
92	Being a second generation immigrant	Individual	13
93	Number of living children in the family	Family/Caregiver	15
94	Urbanicity	Society	12
95	Climate change	Society	11
96	Ethnicity	Individual	11
97	Smoking status of primary caregiver(s)	Family/Caregiver	13
98	Multi-generational families within the same home	Family/Caregiver	13
99	Religiosity of caregiver(s)	Family/Caregiver	13
100	Child younger than classmates	Individual	13
101	Single parent/caregiver family	Family/Caregiver	16
102	Religiosity	Individual	12
103	Not breast fed	Individual	10
104	Month of birth	Individual	12
105	High socioeconomic status family	Family/Caregiver	15
106	Primary caregiver(s) marital status	Family/Caregiver	16

Domain 2: Behavioural

Rank within Domain	Risk Factor	Level	Number of Raters (N)
1	Heavy alcohol use	Individual	14
2	Poor sleep patterns	Individual	14
3	Sexual risk-taking	Individual	14
4	Prescription drug abuse	Individual	11
5	Higher behavioural inhibition	Individual	10
6	Non-prescription drug use	Individual	12
7	Frequent social media use	Individual	14
8	Physical inactivity	Individual	13
9	Unhealthy diet in family/primary caregiver(s)	Family/Caregiver	14
10	Smoking	Individual	13
11	Physical inactivity in family/primary caregiver(s)	Family/Caregiver	14
12	Nitrous oxide use	Individual	8
13	High levels of ultra-processed foods in diet	Individual	8
14	Unhealthy diet	Individual	13
15	Lower behavioural inhibition	Individual	10
16	Foods high in trans fats in diet	Individual	10
17	Excessive sports participation	Individual	12
18	Low sports participation	Individual	12

Rank within Domain	Risk Factor	Level	Number of Raters (N)
19	Diet low in plant-matter diversity	Individual	8
20	Excessive physical activity	Individual	12
21	High ratio of omega-6 to omega-3 fatty acids in diet	Individual	6
22	Low levels of omega-3 polyunsaturated fatty acids in diet	Individual	6
23	Diet low in plant-matter	Individual	9
	**Domain 3: Education and Employment**		
**Rank within Domain**	**Risk Factor**	**Level**	**Number of Raters (N)**
1	School exclusions	Individual	7
2	Quality of school climate (e.g. relating to school connectedness, feelings of safety in school, perception of school, adult-student relationships, morale)	Society	6
=3	Disruptive behaviours in school (e.g. defiance and non-compliance)	Individual	7
=3	Poor peer relationships	Individual	7
=3	Poor school attendance	Individual	7
=3	Poor teacher-pupil relationship	Individual	7
7	Having an Education and Health Care Plan (EHCP)	Individual	7
8	Out-of-school discipline (e.g. suspension and expulsion)	Individual	6
9	Poor school climate (e.g. Ofsted weightings)	Society	6
10	Special Educational Needs (SEN)	Individual	7
11	Poor quality or a lack of social-emotional learning programmes in pre-school	Society	7
=12	Home schooling (not by choice)	Family/Caregiver	7
=12	Negative experience of home schooling	Individual	7
14	Increased pressure/stress for teachers (e.g. insufficient pay/resources and poor leadership)	Society	6
15	Language impairment in primary caregiver(s)	Family/Caregiver	7
16	Poor educational attainment	Individual	7
17	Unemployment of the individual	Individual	6
18	Lack of imitation games with caregivers by age 1	Individual	5
19	Serving in the military	Individual	3
20	Low education level of primary caregiver(s)	Family/Caregiver	7
21	Child not read to daily by age 1	Individual	6
22	School-level deprivation (e.g. proportion eligible for free school meals)	Society	6
23	Participation in the Free-Lunch Program	Individual	6
24	School composition (e.g. size/headcount, gender proportions, ethnicity proportions)	Society	5
25	Low education level of caregiver(s) (other than primary caregiver(s))	Family/Caregiver	6
	**Domain 4: Biomarkers**		
**Rank within Domain**	**Risk Factor**	**Level**	**Number of Raters (N)**
1	Low intelligence quotient (IQ)	Individual	3
2	Genetic markers	Individual	3
3	Sex (biological)	Individual	3
4	Nutritional deficits	Individual	2
5	Neurotoxicity	Individual	3
6	Neuronal atrophy	Individual	2

7 Increased neurodegeneration	Individual	3
8 Reduced neuroplasticity	Individual	2
9 Low levels of dopamine	Individual	2
10 Low levels of nutrients	Individual	2
11 Low levels of serotonin	Individual	2
12 Reduced neurogenesis	Individual	2
13 HPA axis and glucocorticoid receptor resistance	Individual	1
14 Low levels of folate	Individual	1
15 Reduced levels of BDNF (i.e. neurotrophic factor)	Individual	1
16 Reduced grey matter in hippocampus	Individual	2
17 Raised levels of glucocorticoids/cortisol	Individual	2
18 High levels of cytokine proteins (e.g. interleukin-1 [IL-1]; interleukin-6 [IL-6])	Individual	1
19 Birth weight	Individual	3
20 Raised C-reactive protein (CRP)	Individual	2
21 Low levels of vitamin D	Individual	2
22 Decreased neuronal branching	Individual	2
23 Low levels of melatonin	Individual	1
24 Low serum ferritin	Individual	2
25 Changes in prefrontal cortex (e.g. reduced total volume; low myelination; low white matter; increased middle inferior and ventral regions and superior/dorsal regions)	Individual	2
=26 Age of parents at time of giving birth	Individual	3
=26 High intelligence quotient (IQ)	Individual	3
=28 Epigenetic marker - DNA methylation	Individual	1
=28 Epigenetic marker - non-coding ribonucleic acids	Individual	1
28 Gut microbiome (reduced diversity and reduced populations of certain bacteria)	Individual	1
31 Epigenetic marker - chromatin regulation	Individual	1
32 Chronic inflammation	Individual	1
33 Changes in amygdala (e.g. reduced volume)	Individual	2
34 Birth length	Individual	2
_ CD14 levels	Individual	0
_ High levels of indoleamine-2,3-dioxygenase (IDO)	Individual	0
_ High levels of tryptophan-2,3-dioxygenase (TDO)	Individual	0
_ High peripheral manganese	Individual	0
_ High permeability of blood-brain barrier	Individual	0
_ Increased levels of chemokines	Individual	0
_ Increased levels of eicosanoids	Individual	0
_ Increased quinolinic acid (QUIN)	Individual	0
_ Kynurenine/tryptophan ratio	Individual	0
_ Low levels of B vitamins	Individual	0
_ Low or high levels of GDNF (i.e. neurotrophic factor)	Individual	0
_ Low or high levels of Tumour Necrosis Factor alpha (TNF-α)	Individual	0
_ Neutrophil–lymphocyte ratio (NLR)	Individual	0
_ Reduced kynurenic acid (KYNA)	Individual	0

Domain 5: Physical Health			
**Rank within Domain**	**Risk Factor**	**Level**	**Number of Raters (N)**
1	Traumatic brain injury	Individual	6
2	Primary caregiver(s) chronic (long lasting) illness	Family/Caregiver	6
3	Severe health condition	Individual	6
4	Severe illness in family	Family/Caregiver	6
5	Sleep disorder	Individual	6
6	Physical disability	Individual	6
7	Global developmental delay	Individual	6
8	Prolonged duration of a physical health condition	Individual	5
9	Irritable Bowel Syndrome (IBS)	Individual	5
10	Lack of response to treatment for a physical health condition	Individual	4
11	Diabetes	Individual	6
12	Hearing impairment causing disability (e.g. deafness)	Individual	6
13	Autoimmune disorders (e.g. rheumatoid arthritis)	Individual	6
14	Hypoxia (at birth)	Individual	4
15	Maternal obesity/overweight during pregnancy	Individual	2
16	Visual impairment causing disability (e.g. blindness/partial sightedness)	Individual	5
17	Thyroid disease	Individual	5
18	Congenital malformations	Individual	5
19	5-min Apgar score <7	Individual	6
20	Asthma	Individual	6
21	Anaemia	Individual	6
22	Premature birth	Individual	6
=23	Low diversity and composition of gut microbiota	Individual	3
=23	Repeated infections	Individual	5
25	Pre-eclampsia	Individual	4
26	Inflammatory diseases (e.g. Lyme disease)	Individual	5
=27	Maternal hypertension during pregnancy	Individual	1
=27	Maternal infection requiring hospitalisation during pregnancy	Individual	1
=27	Maternal diabetes during pregnancy	Individual	1
=27	Chronic (long lasting) infection (e.g. Lyme disease, periodontal disease)	Individual	5
=31	Low serum vitamin D	Individual	5
=31	Maternal auto-immune disease during pregnancy	Individual	1
33	Perinatal infections (e.g. cytomegalovirus)	Individual	6
34	Family history of autoimmune disease	Family/Caregiver	6
35	Chronic (long lasting) gastric ill-health (e.g. inflammatory bowel disease)	Individual	5
36	Obstetric complications (other)	Individual	4
37	Disease history	Individual	3
38	Dental caries (tooth decay)	Individual	6
39	Chronic (long lasting) reflux or indigestion	Indiivdual	5
40	Allergies (e.g. non-IgE-mediated food allergies, pollen allergies)	Individual	6

41	Family history of psoriasis	Family/Caregiver	4
42	Obesity/overweight	Individual	4
43	Body Mass Index (BMI)	Individual	5
44	Eczema	Individual	6
45	Long term antibiotic use	Individual	4
46	Family history of Type 1 diabetes	Family/Caregiver	6
47	Family history of rheumatoid arthritis	Family/Caregiver	4
48	Maternal self-rated health (prior to pregnancy)	Family/Caregiver	4
49	Dental erosion	Individual	4
50	Polyhydramnios	Individual	3
51	Ruptured membranes (at birth)	Individual	4
_	Maternal acetaminophen (paracetamol) use during pregnancy	Individual	0

Domain 6: Psychological and Mental Health

Rank within Domain	Risk Factor	Level	Number of Raters (N)
1	Family history of severe mental illness (e.g. psychosis)	Family/Caregiver	14
2	Primary caregiver(s) mental health problems	Family/Caregiver	14
3	Psychiatric history	Individual	14
4	Family history of psychiatric disorders	Family/Caregiver	14
5	Social-communication problems	Individual	14
6	Neuro-developmental conditions (e.g. autism)	Individual	13
7	Increased panic attacks	Individual	13
8	Anxiety (as a predictor of further mental health problems)	Individual	14
=9	High levels of perceived stress	Individual	13
=9	Ineffective coping strategies	Individual	13
11	Maternal psychosis (perinatal or pre-natal)	Individual	12
12	Lack of psychological resilience	Individual	14
13	Attention (as a predictor of further mental health problems)	Individual	13
14	Intellectual disability	Individual	12
15	Increased anxiety arousal	Individual	13
16	Dissociation during traumatic experience	Individual	12
17	Acute stress symptoms (anxiety, avoidance or depression - as a predictor of further mental health problems)	Individual	14
18	Acute stress disorder (as a predictor of further mental health problems)	Individual	14
19	Excessive rumination	Individual	13
20	Poor concentration	Individual	13
21	Negative attitudes to mental health problems within society an individual lives in	Society	14
22	Maternal depression during pregnancy	Individual	12
23	Poor problem-solving abilities	Individual	13
24	Emotional reactivity	Individual	14
25	Poor visuospatial functioning	Individual	10
26	Problems with memory	Individual	13

Domain 7: Patterns of Service Use			
**Rank within Domain**	**Risk Factor**	**Level**	**Number of Raters (N)**
1	Child protection record	Individual	6
2	Investigations by multiple services suggestive of suffering from medically unexplained symptoms	Individual	7
3	Failure to attend three or more planned health or social care appointments	Individual	7
4	Three or more presentations to emergency services within a year	Individual	6
5	Repeat hospitalisation	Individual	6
6	Missed ante-natal visits	Individual	6
7	Primary caregiver(s) non-attendance at baby groups	Family/Caregiver	7

Domain 8: Factors Identified to be Particularly Relevant to Under-Served Populations			
**Rank within Domain**	**Risk Factor**	**Level**	**Number of Raters (N)**
1	Being a looked after child (LAC)	Individual	5
2	Child in need (CIN) status	Individual	5
3	Torture	Individual	5
4	Separation from family (e.g. out-of-home care)	Individual	6
5	Child protection record	Individual	6
6	Heavy alcohol use	Individual	6
7	Victim of organised crime (e.g. commercial sexual exploitation or via county lines)	Individual	5
8	Imprisonment	Individual	5
9	Homelessness (young person has left home)	Individual	6
10	Primary caregiver(s) mental health problems	Family/Caregiver	6
11	Victim or witness of violent crime	Individual	6
12	Experience of bereavement during a traumatic event	Individual	6
13	Victim of structural/systemic racism	Society	6
14	Being an asylum-seeking family	Family/Caregiver	6
15	Homelessness	Family/Caregiver	6
16	Household mental illness	Family/Caregiver	6
17	War/conflict	Society	6
=18	Gangland crime	Society	6
=18	Family experience of social exclusion, discrimination and harassment associated with ethnicity	Family/Caregiver	6
20	Witnessing injury/death during a traumatic event	Individual	6
21	Neuro-developmental conditions (e.g. autism)	Individual	6
22	Experience of racism and discrimination	Individual	6
23	Disabled child/young person experiencing infantilisation	Individual	2
24	Out-of-school discipline (e.g. suspension and expulsion)	Individual	6
25	Being an unaccompanied asylum seeker	Individual	6
26	Famine	Society	5
=27	Household drug abuse	Family/Caregiver	6
=27	Household member involvement in criminal justice system	Family/Caregiver	6
29	Being a young carer	Individual	6

30	Radicalisation	Society	6
31	Organised crime affiliation	Individual	5
32	Household alcohol abuse	Family/Caregiver	6
33	Involvement in criminal justice system	Individual	6
34	Witness of community violence	Individual	6
35	School exclusions	Individual	6
36	Family has low trust in services	Family/Caregiver	6
37	Household criminality	Family/Caregiver	6
38	Imprisonment of household member	Family/Caregiver	5
39	Non-prescription drug use	Individual	6
40	Poor school attendance	Individual	6
41	Maternal substance abuse during pregnancy	Individual	6
42	Poor school climate (e.g. Ofsted weightings)	Society	6
43	Being an adoptee	Individual	5
44	Cultural racism	Society	6
45	Prescription drug abuse	Individual	6
46	Having an Education and Health Care Plan (EHCP)	Individual	5
47	Failure to attend three or more planned health or social care appointments	Individual	6
48	Maternal use of psychotropics during pregnancy	Individual	5
49	Maternal alcohol use during pregnancy	Individual	6
50	Ethnic minority in low ethnic density area	Society	6
51	Special Educational Needs (SEN)	Individual	6
52	Quality of school climate (e.g. relating to school connectedness, feelings of safety in school, perception of school, adult-student relationships, morale)	Society	6
53	Poverty	Family/Caregiver	6
54	Poor educational attainment	Individual	6
55	Intellectual disability	Individual	6
=56	Poor quality or a lack of social-emotional learning programmes in pre-school	Society	5
=56	Maternal depression during pregnancy	Individual	5
58	Physical disability	Individual	6
59	Maternal psychosis (perinatal or pre-natal)	Individual	5
60	Negative previous experiences of services	Individual	6
61	Being a young parent	Individual	6
62	Household overcrowding	Family/Caregiver	6
63	Family financial problems	Family/Caregiver	6
64	Area deprivation (area code)	Society	6
65	Food poverty	Family/Caregiver	6
66	Minority gender identity	Individual	4
67	Racial bias in obstetric care	Society	5
68	School-level deprivation (e.g. proportion eligible for free school meals)	Society	6
69	Personal direct experience of natural disaster	Individual	6
70	Living in an area with high crime levels	Society	6
71	Increased pressure/stress for teachers (e.g. insufficient pay/resources and poor leadership)	Society	6
72	Language impairment in primary caregiver(s)	Family/Caregiver	6
73	Unemployment of the individual	Individual	6

74	Trapped during earthquake	Individual	5
75	Minority sexuality	Individual	5
76	Primary caregiver(s) parenting styles (strict/rigid/conventional)	Family/Caregiver	6
77	Hearing impairment causing disability (e.g. deafness)	Individual	6
78	Primary caregiver(s) unemployment	Family/Caregiver	6
79	Social-communication problems	Individual	6
80	Air pollution	Society	6
81	Participation in the Free-Lunch Program	Individual	6
82	Low education level of primary caregiver(s)	Family/Caregiver	6
83	School composition (e.g. size/headcount, gender proportions, ethnicity proportions)	Society	4
84	Multi-generational families within the same home	Family/Caregiver	6
85	Migration from another country	Family/Caregiver	6
86	Visual impairment causing disability (e.g. blindness/partial sightedness)	Individual	5
=87	Serving in the military	Individual	3
=87	Missed ante-natal visits	Individual	5
89	English as an additional language	Society	6
90	Nitrous oxide use	Individual	2
91	Digital exclusion	Individual	4
92	Low socioeconomic status	Family/Caregiver	6
93	Remoteness of living	Society	6
94	Low education level of caregiver(s) (other than primary caregiver(s))	Family/Caregiver	6
95	Urbanicity	Society	5
96	Ethnicity	Individual	5
97	Climate change	Society	5
98	Belonging to a religious minority	Family/Caregiver	6
99	Belonging to a traveller community	Family/Caregiver	5
100	Religiosity	Individual	5
101	Religiosity of caregiver(s)	Family/Caregiver	6
102	Being a second generation immigrant	Individual	6

Key:

= = Tied positions

_ = Risk factor has no ranking because it was unable to be rated by any respondents
